# Unmatched Cell Line Collections Are Not Optimal for Identification of PARP Inhibitor Response and Drug Synergies

**DOI:** 10.1111/jcmm.70845

**Published:** 2025-09-22

**Authors:** Zoe Phan, Kristine J. Fernandez, C. Elizabeth Caldon

**Affiliations:** ^1^ St Vincent's Healthcare Clinical Campus, School of Clinical Medicine Sydney New South Wales Australia; ^2^ Garvan Institute of Medical Research New South Wales Australia

**Keywords:** cell line, chemotherapy, combination therapy, PARP inhibitors

## Abstract

PARP inhibitors show great efficacy in *BRCA1*/*2‐*mutated patients, but many preclinical studies, including combination therapies, fail to translate clinically, likely due to the limitations of preclinical models. This brief report aimed to identify appropriate cell line models to investigate PARP inhibitor sensitivity and synergies. An *in silico* study of cell line collections was performed to assess the correlation between *BRCA1/2* mutations and sensitivity to PARP inhibitor monotherapy or with platinum‐based chemotherapy. Subsequently, we characterised an isogenic model containing *Brca1* and *Brca2* mutations and investigated treatment response. Using cell line collections, *BRCA1‐* and *BRCA2‐*altered cell lines were not associated with increased sensitivity to PARP inhibitors. Other factors, including high *PARP1* expression and low‐level genome alterations, showed correlation with increased sensitivity to a PARP inhibitor. Furthermore, cell line collections did not reflect the improved patient outcomes arising from combination PARP inhibitor and platinum‐based chemotherapy. In contrast, the ID8 isogenic model, with specific *Brca1* and *Brca2* mutations, reflected patient tumour‐like responses to PARP inhibitor monotherapy and combination therapy. This study suggests exercising caution when using cell line collections as part of model selection when investigating PARP inhibitor sensitivity and synergy. Our data propose that using an isogenic preclinical model is more likely to accurately reflect patient tumour response. However, as this study was limited to a single isogenic model, further validation in additional systems will be required to broaden the scope of these observations.

In the clinic, patients with a *BRCA1* or *BRCA2* mutation derive a significant benefit from PARP inhibitor treatment, a finding which has been confirmed in dozens of clinical trials across multiple cancer types including ovarian, breast and prostate cancers [1, 2, 3]. Given the current success, but limited use, of PARP inhibitors, appropriate pre‐clinical models are required to develop new applications for PARP inhibitors. Of particular difficulty is extending the use of PARP inhibitors to identify where they can successfully be used in combination with other therapies. While there have been many combination therapy trials and preclinical studies, there are only a handful of approvals (e.g., talazoparib and enzalutamide in metastatic prostate cancer [4], and bevacizumab and olaparib in ovarian cancer [5]). The failure of many preclinical studies to translate to clinical approval is potentially due to the limitations of current preclinical models, confounded by the extensive genetic heterogeneity in established cell lines [6, 7]. Therefore, in this brief report, we aimed to identify appropriate cell line models suitable for both PARP inhibitor sensitivity and synergy studies.

Firstly, we assessed the benefit of cell line collections in signal seeking PARP inhibitor studies by systematically analysing cell lines from the Cancer Cell Line Encyclopedia (Broad, 2019) [8, 9, 10], accessed via cBioPortal. We extracted information on *BRCA1* and *BRCA2* mRNA expression, mutation status, and copy number alterations, alongside IC_50_ values for PARP inhibitor monotherapies. The list of cell lines alongside *BRCA1* and *BRCA2* mutation status is found in Tables S1 and S2.

Surprisingly, in *BRCA1*‐mutant cell lines, *BRCA1* mRNA expression showed no correlation with IC_50_ response to olaparib (*r* = −0.27, *p* = 0.42) (Figure 1A; left). Counterintuitively, among *BRCA1*‐wildtype cell lines, lower *BRCA1* mRNA expression was significantly associated with reduced sensitivity to olaparib, as indicated by the significant negative correlation (*r* = −0.20, *p* < 0.0001) (Figure 1A; left). This was unexpected, given that *BRCA1*‐mutant cell lines exhibited lower *BRCA1* mRNA expression than *BRCA1‐*wildtype cell lines (Figure S1A).

**FIGURE 1:**
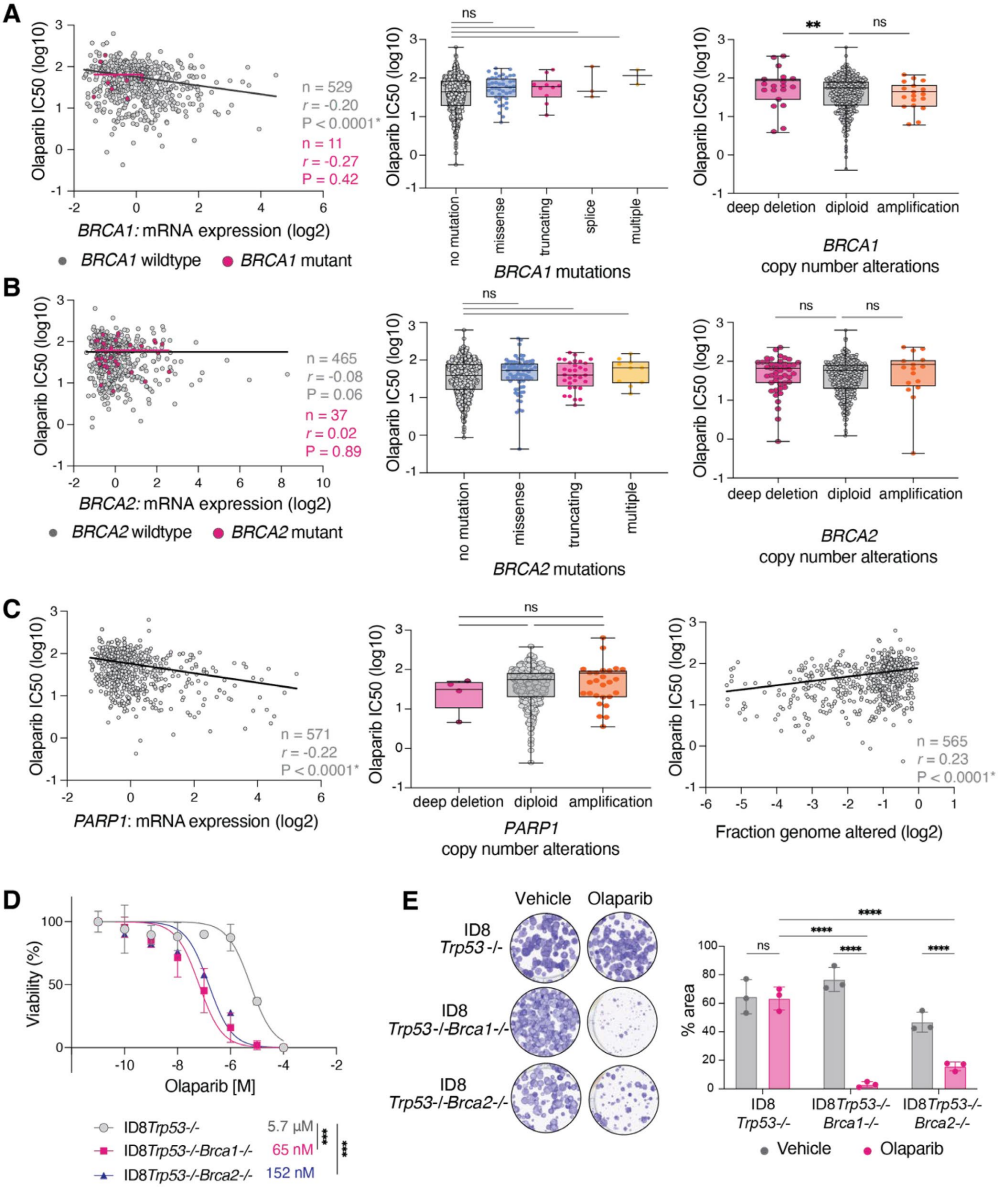
Model selection is critical for detecting changes in PARP inhibitor sensitivity due to *BRCA1/BRCA2* mutations. (A, B) Analysis of cell line databases demonstrates that (A) *BRCA1*‐mutant and (B) *BRCA2*‐mutant cell lines across different cancer types do not fully represent patient tumour responses to olaparib. Left panels: Correlation between olaparib IC_50_ response and *BRCA1/BRCA2* mRNA expression relative to diploid samples. Pink dots indicate cell lines with predicted driver mutations in *BRCA1* (*n* = 11) and *BRCA2* (*n* = 37), while grey dots indicate wildtype *BRCA1* (*n* = 529) and *BRCA2* (*n* = 465) cell lines. Best‐fit linear regressions (pink and black lines) illustrate positive or negative correlations. Middle panels: Olaparib IC_50_ response in *BRCA1‐* and *BRCA2‐*mutant cell lines with different mutation types: No mutation (*n* = 810 for *BRCA1*, *n* = 752 for *BRCA2*), missense (*n* = 45, *n* = 76), truncating (*n* = 10, *n* = 32), splice (*n* = 3) and multiple (*n* = 2, *n* = 10). Right panels: Olaparib IC_50_ response in cell lines with different *BRCA1/BRCA2* copy number alteration statuses. IC_50_ values are shown for cell lines classified as deep deletion (*n* = 19 for *BRCA1*, *n* = 47 for *BRCA2*), diploid (*n* = 518, *n* = 492), and amplification (*n* = 18, *n* = 16). (C) High *PARP1* expression and low genome alterations are associated with increased olaparib sensitivity in cell line collections. Left panel: Correlation between olaparib IC_50_ response and *PARP1* mRNA expression relative to diploid samples (*n* = 571). Middle panel: Olaparib IC_50_ response in cell lines with *PARP1* copy number alterations classified as deep deletion (*n* = 4), diploid (*n* = 525) and amplification (*n* = 26). Right panel: Correlation between olaparib IC_50_ response and fraction genome alterations (*n* = 565). Statistical analyses were determined by two‐sided Pearson's correlation test (left panels) and one‐way ANOVA (middle and right panels). Data were accessed via cBioPortal. (D, E) ID8 isogenic models are optimal for investigating PARP inhibitor sensitivity. (D) Cell viability assays demonstrate that ID8*Trp53−/‐Brca1−/−* and ID8*Trp53−/‐Brca2−/−* cells are more sensitive to olaparib compared to ID8 *Brca* wildtype cells. Cells were treated with a range of olaparib doses for 3 days and IC_50_ values were determined using alamarBlue assay (*n* = 3 biological replicates). Some error bars are not visible as they are smaller than the data points. (E) Colony forming assays of ID8 cells treated with vehicle (DMSO) or 1 μM olaparib for 48 h demonstrate a decrease in colony formation with olaparib treatment in *Brca*‐mutated cell lines. Statistical analyses across cell lines were determined by two‐way ANOVA. Statistical analyses for comparison within cell lines were determined by two‐sided *t*‐test. *N* = 3 biological replicates. **p < 0.01; ***p < 0.001; ****p < 0.0001; ns = non‐significant.

Next, we examined whether specific types of mutation influence PARP inhibitor response. We found no significant difference in IC_50_ values between *BRCA1*‐mutant cell lines (missense, truncating, splice or multiple mutations) and *BRCA1‐*wildtype cell lines (Figure 1A; middle). We acknowledge that certain subgroup analyses (splice and multiple) were based on small sample sizes (*n* = 2–3), which limits statistical power. Nonetheless, even cell lines harbouring predicted driver mutations failed to exhibit increased sensitivity to PARP inhibitors compared to those without *BRCA1* mutations (Figure S1C). Contrary to expectations, cell lines with a *BRCA1* deep deletion demonstrated reduced sensitivity to olaparib compared to those with normal *BRCA1* copy number (*p* = 0.01) (Figure 1A; right). This finding is particularly intriguing, given that *BRCA1* deep deletion is associated with lower *BRCA1* mRNA levels (Figure S1B).

Analysis of *BRCA2*‐alterated cell lines revealed similar trends to those observed in *BRCA1*‐altered cell lines. In *BRCA2*‐mutant cell lines, *BRCA2* mRNA expression did not correlate with olaparib response (*r* = 0.02, *p* = 0.89) (Figure 1B; left). In *BRCA2*‐wildtype cell lines, there was a significant negative correlation between mRNA expression and treatment response (*r* = −0.08, *p* = 0.06) (Figure 1B; left). There was no association between *BRCA2* mutation type and olaparib response (Figure 1B; middle). Similarly, *BRCA2* deep deletion did not alter olaparib sensitivity compared to diploid cell lines, despite being associated with lower *BRCA2* mRNA expression (Figure 1B; right) (Figure S1B).

We further analysed the impact of *BRCA1* and *BRCA2* alterations on the response to additional PARP inhibitors, including talazoparib, rucaparib, veliparib, as well as the platinum‐based chemotherapy, cisplatin (Figures S2 and S3). Across all analyses, *BRCA1‐* or *BRCA2‐*mutant or deep‐deleted cell lines did not demonstrate increased sensitivity to PARP inhibitors or platinum‐based chemotherapy. Furthermore, subgroup analyses using only ovarian cancer cell lines, representing the most clinically responsive group, showed no association between *BRCA1* and *BRCA2* alterations and response to olaparib (Figure S4).

Given that *BRCA1* and *BRCA2* mutations in cell line collections did not predict PARP inhibitor response, we explored alternative biomarkers that may better correlate with sensitivity. Notably, high *PARP1* mRNA expression was significantly associated with increased responsiveness to olaparib across all cell lines (*r* = −0.22; *p* < 0.0001) (Figure 1C; left). However, *PARP1* copy number alterations (deletion or amplification) did not impact olaparib response (Figure 1C; middle).

Another predictive factor was the fraction genome altered in each cell line, where cell lines with fewer genomic alterations were more sensitive to olaparib (*r* = 0.23, *p* < 0.0001) (Figure 1C; right). Together, these findings highlight that in cell lines, both high *PARP1* gene expression and low‐level genome alterations are stronger predictors of olaparib response than *BRCA* alterations (Figure 1A/B versus C).

Given that cell line collections are not representative of *BRCA1‐* and *BRCA2‐*altered patient tumour response to PARP inhibitors, we hypothesised that an isogenic model hosting specific gene alterations would be more appropriate to explore cellular responses to PARP inhibitor treatment. Therefore, we characterised the ID8 model of ovarian cancer, with matched cell lines with specific mutations in *Brca1* and *Brca2* [11, 12]. ID8*Trp53−/‐Brca1−/−* and ID8*Trp53−/‐Brca2−/−* cells were significantly more sensitive to olaparib than ID8*Trp53−/‐Brca‐*wildtype cells by measuring cell viability (Figure 1D). Furthermore, cells with *Brca1* or *Brca2* mutation demonstrated a significant decrease in the ability to form colonies in the presence of olaparib compared to *Brca*‐wildtype cells (Figure 1E). Thus, the ID8 model with specific mutations in *Brca1* and *Brca2* is significantly more sensitive to olaparib compared to their *Brca‐*wildtype counterpart, in line with previous observations [11, 12, 13].

In a previous study, using clinical data, we demonstrated that *BRCA1/2*‐mutated patient tumours respond better to combination PARP inhibition and chemotherapy compared to *BRCA*‐wildtype patient tumours [6]. Therefore, we assessed whether cell line collections with a *BRCA1/2* mutation recapitulate this response observed in the clinic by analysing synergy data from the Genomics Drug Sensitivity in Cancer drug combination database (GDSC; Wellcome Sanger Institute) [14]. Based on the GDSC, a drug combination is deemed synergistic when ΔIC_50_ ≥ 3 or the ΔEMax ≥ 0.2. Specifically, ΔIC_50_ is used to measure shifts in potency, while ΔEMax indicates shifts in efficacy [14].

We examined the clinically effective combination of PARP inhibitor and platinum‐based chemotherapy in cell lines with a predicted driver mutation in *BRCA1* or *BRCA2* compared to *BRCA1/BRCA2* wildtype cell lines [15, 16, 17, 18, 19, 20]. When investigating synergy between olaparib and cisplatin, there was no significant difference in ΔIC_50_ or ΔEMax based on mutation status (Figure 2A). Furthermore, these values did not cross ΔIC_50_ ≥ 3 and ΔEMax ≥ 0.2, and therefore, were deemed antagonistic. Although subgroup analyses of synergy in *BRCA1‐*mutant cell lines (Figure 2A) were based on small sample sizes (*n* = 3), which may limit statistical power, the overall trend remained consistent with our broader findings.

**FIGURE 2:**
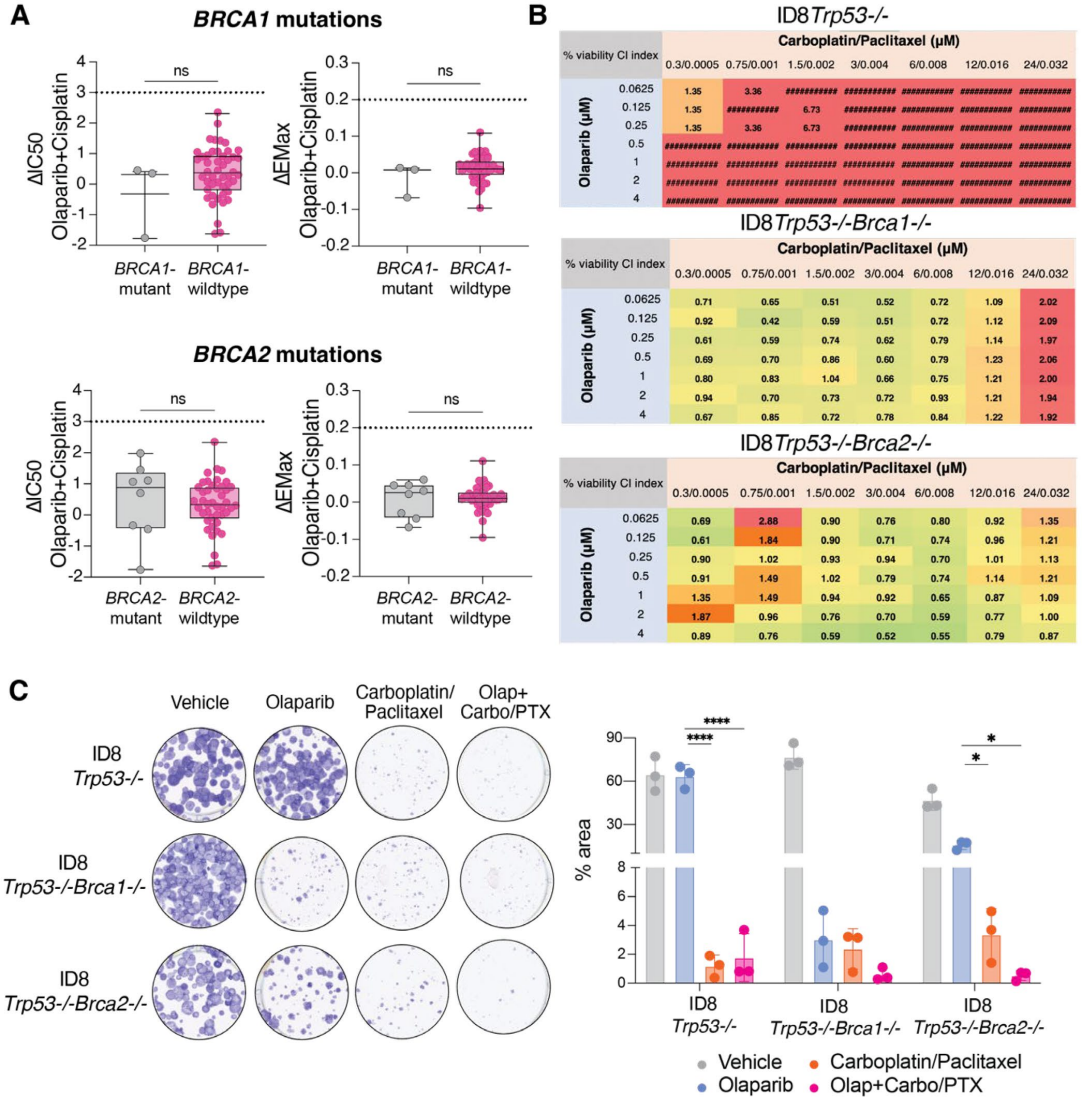
Isogenic models with specific *BRCA1/BRCA2* mutations are suitable for investigating PARP inhibitor drug synergies. (A) Analysis of breast cancer cell line collections shows no significant association between *BRCA1/BRCA2* mutation status and sensitivity to combined PARP inhibitor and chemotherapy compared to *BRCA*‐wildtype. ΔIC_50_ and ΔEMax drug combination response for olaparib + cisplatin are shown for breast cancer cell lines with a *BRCA1* (*n* = 3) or *BRCA2* mutation (*n* = 8) and *BRCA‐*wildtype (*n* = 47 for *BRCA1*, *n* = 43 for *BRCA2*). Synergism is defined as ΔEMax ≥ 0.2 and ΔIC_50_ ≥ 3, where ΔIC_50_ is defined as a shift in potency and ΔEMax as shift in efficacy (more details in Data S1). Statistical analyses were performed using an unpaired two‐sided *t*‐test. Lines represent the median and interquartile range. Data were accessed from the Genomics of Drug Sensitivity in Cancer Drug Combinations dataset [14]. (B) Combination PARP inhibition and chemotherapy is synergistic in isogenic *Brca‐*mutated but not in *Brca‐*wildtype cells. ID8*Trp53−/−*, ID8*Trp53−/‐Brca1−/−*, ID8*Trp53−/‐Brca2−/−* cells were treated with doses of olaparib and carboplatin/paclitaxel for 3 days with vehicle‐treated cells as controls. Cell viability was measured using alamarBlue. Synergy analysis was performed using Compusyn, generating a combination index (CI) value where green (< 1) indicates synergism, yellow (=1) indicates additivity and red (> 1) indicates antagonism. Data are pooled from three biological replicates. (C) Colony forming assays of ID8 cells treated with vehicle (DMSO), 1 μM olaparib, 6 μM carboplatin/8 nM paclitaxel (olap+carbo/PTX) for 7 days. Quantification of colony formation is presented as % area normalised to vehicle within each cell line. Statistical analyses were performed using one‐way ANOVA. *p < 0.05; ****p < 0.0001; ns = non‐significant.

Subsequently, we investigated whether an isogenic model would be suitable to study the effects of PARP inhibitor combination therapies. From assessing synergy, the combination of olaparib and carboplatin/paclitaxel (standard‐of‐care in ovarian cancer) was antagonistic in *Brca‐*wildtype cells (ID8*Trp53−/−*), while the combination therapy was synergistic in ID8*Trp53−/−Brca1−/−* and ID8*Trp53−/−Brca2−/−* cells (Figure 2B). Moreover, the combination therapy was effective in reducing colony growth in *Brca2−/−* cell lines (Figure 2C).

Taken together, this brief report highlighted the importance of exercising caution when using cell line collections in model selection when investigating PARP inhibitor sensitivities and synergies. These data further suggest using isogenic models with specific mutations in *BRCA1* and *BRCA2* and to potentially avoid comparing cell lines from diverse genetic backgrounds, as they may not accurately reflect patient tumour responses.

During this study, Takamatsu et al. published a study confirming that cell lines with alterations in *BRCA1* and *BRCA2* had no association with response to PARP inhibitors and platinum agents, as measured by area under the curve (AUC) analysis [21]. Using additional measures, such as ‘HRD score’ and mutational ‘Signature 3’, they confirmed that changes associated with homologous recombination deficiency did not demonstrate increased response to PARP inhibitors [21]. Additional studies found that signatures related to homologous recombination deficiency had poor predictive power of PARP inhibitor response in colorectal cancer cell lines [22], and *BRCA* status was not predictive of therapy response in a small panel of ovarian cancer cell lines [23]. Our collective findings suggest that cell line collections, with diverse genetic backgrounds, do not accurately reflect patient tumour response to PARP inhibitors. Here, we show that this finding extends to synergy studies in that *BRCA1/BRCA2* status in cell line panels is unable to accurately predict the combination effects of PARP inhibitors with chemotherapy.

While we found a positive correlation between *PARP1* expression and response to PARP inhibitors, which is in line with many in vitro studies [24, 25], in patient tumours, the relationship between *PARP1* expression and sensitivity to PARP inhibitors is more complicated. Although adequate PARP1 protein is necessary for PARP inhibitor function, it is not clear whether high *PARP1* expression increases sensitivity to PARP inhibitors in tumours. Most clinical trials do not investigate *PARP1* expression, although *PARP1* expression correlated with uptake of PARP inhibitors in a small phase I trial [26]. Finally, we also revealed that cell lines with lower genome complexity were a better predictor of response to PARP inhibitors than *BRCA* mutations. This suggests that preclinical models with high *PARP1* expression and a simpler genomic profile may be more conducive to identifying clinically translatable effects of PARP inhibitors.

We found that isogenic models with specific mutations in *Brca1* and *Brca2* recapitulated patient sensitivity to PARP inhibitors. These are in line with multiple in vitro studies demonstrating the sensitivity of ID8*Trp53−/‐Brca1−/−* and ID8*Trp53−/‐Brca2−/−* cells to PARP inhibitors, including the original studies that generated the mutant models [11, 12, 13]. These data translate to the in vivo setting, where ID8*Trp53−/‐Brca1−/−* and ID8*Trp53−/‐Brca2−/−* tumour‐bearing mice demonstrated increased survival after rucaparib treatment [13]. Furthermore, our data validate that ID8 cells with *Brca* mutations recapitulate patient response to combination PARP inhibitor and chemotherapy [6]. While we propose that the ID8 model is helpful, particularly due to its ease of use, it is important to note that we utilised a single isogenic cell line. However, these findings have also been observed in other models, such as RPE‐1 [27]. Moreover, while the ID8 model is syngeneic and allows for use in immunocompetent mice, there are inherent differences in the physiology between mice and humans that may confound the interpretation of preclinical data. Therefore, validating these findings in a 2D representative human model, such as OVCAR‐4 [28], or 3D organoid systems, would help ensure the validity of the results.

Overall, we conclude that standard cell line collections are not the optimal tool to draw conclusions on the action of PARP inhibitors or developing new clinical applications. We have shown that PARP inhibitor and chemotherapy combination synergises to induce reduced viability on an isogenic background, representative of the improved outcomes seen in patients. Reverse engineering of other successful combinations applied in patients (e.g., VEGF inhibitors and PARP inhibitors) could further delineate the most appropriate models for PARP inhibitor research [29].

## Author Contributions

**Zoe Phan:** data curation (lead), formal analysis (lead), investigation (lead), writing – original draft (lead). **Kristine J. Fernandez:** data curation (equal). **C. Elizabeth Caldon:** conceptualization (lead), funding acquisition (lead), supervision (lead), writing – review and editing (lead).

## Conflicts of Interest

The authors declare no conflicts of interest.

## Supporting information


**Table S1:** List of cell lines from the Cancer Cell Line Encyclopedia with predicted *BRCA1* driver mutation status. Cell lines with mutations but of variance of unknown significance (VUS) were excluded from analysis. Cell lines with no mutation were considered ‘wildtype’. Driver annotations were based on OncoKB and Hotspots from cBioPortal.
**Table S2:** List of cell lines from the Cancer Cell Line Encyclopedia with predicted *BRCA2* driver mutation status. Cell lines with mutations but of variance of unknown significance (VUS) were excluded from analysis. Cell lines with no mutation were considered ‘wildtype’. Driver annotations were based on OncoKB and Hotspots from cBioPortal.

**Figure S1:** BRCA1 and BRCA2 mRNA expression levels and IC_50_ response in cell lines with BRCA alterations. (A) BRCA1 and BRCA2 mRNA expression in wildtype (*n* = 529 for BRCA1, *n* = 465 for BRCA2) and mutant (*n* = 11 for BRCA1, *n* = 37 for BRCA2) cell lines. (B) Cell lines with BRCA1 and BRCA2 deep deletions exhibit lower mRNA expression levels. BRCA1 expression is shown for deep deletion (*n* = 27) and diploid (*n* = 863) cell lines, while BRCA2 expression is shown for deep deletion (*n* = 84) and diploid (*n* = 818) cell lines. Statistical analyses were performed using an unpaired *t*‐test. (C) IC_50_ response of various PARP inhibitors (olaparib, talazoparib, rucaparib, veliparib) and cisplatin in BRCA1‐mutant cell lines with predicted driver mutations (*n* = 17–18), variance of unknown significance (VUS) (*n* = 43–48), and no documented mutations (*n* = 809–874). (D) IC_50_ response to the same treatments in BRCA2‐mutant cell lines with predicted driver mutations (*n* = 40–43), VUS (*n* = 75–81), and no documented mutations (*n* = 752–819). Statistical analyses were performed using one‐way ANOVA. *p < 0.01; **p < 0.001; **** *p* < 0.0001; ns = non‐significant. Data were accessed via cBioPortal.
**Figure S2:** Altered BRCA1 expression is not associated with an increase in PARP inhibitor and platinum‐based chemotherapy sensitivity in cell lines. (A‐D) IC_50_ responses to PARP inhibitors (olaparib, talazoparib, rucaparib, veliparib) and cisplatin in cell lines with BRCA1 alterations. Left panels: Correlation between IC_50_ response and BRCA1 expression relative to diploid samples. Red dots indicate cell lines with predicted driver mutations in BRCA1 (*n* = 10–12), while grey dots indicate BRCA1 wildtype (*n* = 528–611) cell lines. Best‐fit linear regressions (red and black lines) illustrate positive or negative correlations. Middle panels: IC_50_ responses in BRCA1‐mutant cell lines with different mutation types: no mutation (*n* = 809–872), missense (*n* = 46–50), truncating (*n* = 10–11), splice (*n* = 3) and multiple (*n* = 2). Right panels: IC_50_ response in cell lines with different BRCA1 copy number alteration statuses. IC_50_ values are shown for cell lines classified as deep deletion (*n* = 19–23), diploid (*n* = 517–563), and amplification (*n* = 19–21). Statistical analyses were determined by two‐sided Pearson's correlation test (left panel) and one‐way ANOVA (middle and right panels). * *p* < 0.05; ** *p* < 0.01; ns = non‐significant. Data were accessed via cBioPortal.
**Figure S3:** Altered BRCA2 expression is not associated with an increase in PARP inhibitor and platinum‐based chemotherapy sensitivity in cell lines. (A‐D) IC_50_ responses to PARP inhibitors (olaparib, talazoparib, rucaparib, veliparib) and cisplatin in cell lines BRCA2 alterations. Left panels: Correlation between IC_50_ response and BRCA2 expression relative to diploid samples. Red dots indicate cell lines with predicted driver mutations in BRCA2 (*n* = 10–12), while grey dots indicate cell lines wildtype BRCA2 (*n* = 528–611) cell lines. Best‐fit linear regressions (red and black lines) illustrate positive or negative correlations. Middle panels: IC_50_ responses in BRCA1‐mutant cell lines with different mutation types: no mutation (*n* = 752–819), missense (*n* = 76–82), truncating (*n* = 30–32), and multiple (*n* = 9–10). Right panels: IC_50_ response in cell lines with different BRCA2 copy number alteration statuses. IC_50_ values are shown for cell lines classified as deep deletion (*n* = 47–53), diploid (*n* = 492–534), and amplification (*n* = 16–19). Statistical analyses were determined by two‐sided Pearson's correlation test (left panel) and one‐way ANOVA (middle and right panels). * *p* < 0.05; *** *p* < 0.001; **** *p* < 0.0001; ns = non‐significant. Data were accessed via cBioPortal.
**Figure S4:** BRCA1 and BRCA2 altered ovarian cancer cell line collections are not associated with an increased sensitivity to a PARP inhibitor. (A, B) IC_50_ responses to olaparib in ovarian cancer cell lines with (A) BRCA1 and (B) BRCA2 alterations. Left panels: Correlation between IC_50_ olaparib response and BRCA1/BRCA2 alterations relative to diploid samples. Red dots indicate cell lines with predicted driver mutations in BRCA1 (*n* = 3) and BRCA2 (*n* = 2), while grey dots indicate wildtype BRCA1 (*n* = 26) and BRCA2 (*n* = 27) cell lines. Best‐fit linear regression (red and black lines) illustrates positive or negative correlations. Linear regression for BRCA2‐mutant ovarian cancer cell lines were unable to be calculated with small sample size. Middle panels: Olaparib IC_50_ response in BRCA1‐ and BRCA2‐mutant cell lines with different mutation types: no mutation (*n* = 34 for BRCA1, *n* = 33 for BRCA2), truncating (*n* = 4 for BRCA1, *n* = 2 for BRCA2), splice (*n* = 1 for BRCA1) and missense (*n* = 4 for BRCA2). Right panels: Olaparib IC_50_ response in ovarian cell lines with different BRCA1/2 copy number alteration statuses. IC_50_ values are shown for cell lines classified as deep deletion (*n* = 3 for BRCA1, *n* = 1 for BRCA2), diploid (*n* = 22 for BRCA1, *n* = 26 for BRCA2) and amplification (*n* = 3 for BRCA1, *n* = 1 for BRCA2). Comparisons for BRCA2 could not be calculated due to small sample size. Where statistical analyses could be determined, they were by two‐sided Pearson's correlation test (left panels) and one‐way ANOVA (middle and right panels). (C, D) mRNA expression of (C) BRCA1 and (D) BRCA2 in ovarian (*n* = 63 for BRCA1, *n* = 26 for BRCA2) and non‐ovarian cancer (*n* = 508 for BRCA1, *n* = 508 for BRCA2) cell lines. Statistical analyses were determined by unpaired *t*‐test. * *p* < 0.05; ns = non‐significant. Data were accessed via cBioPortal.

**Data S1:** Supporting Information.

## Data Availability

The data that support the findings of this study are available in the Cancer Cell Line Encyclopedia, accessed via cBioPortal (https://www.cbioportal.org/) and the Genomics Drug Sensitivity in Cancer database (https://gdsc‐combinations.depmap.sanger.ac.uk/).
