# Supplementary material for: Unmatched Cell Line Collections Are Not Optimal for Identification of PARP Inhibitor Response and Drug Synergies
Source: J Cell Mol Med. 2025 Sep 22;29(18):e70845. doi: 10.1111/jcmm.70845 (PMC12451393; doi:10.1111/jcmm.70845)
Supplement: Supplementary file 1 — Table S1: List of cell lines from the Cancer Cell Line Encyclopedia with predicted BRCA1 driver mutation status. Cell lines with mutations but of variance of unknown significance (VUS) were excluded from analysis. Cell lines with no mutation were considered ‘wildtype’. Driver annotations were based on OncoKB and Hotspots from cBioPortal. Table S2: List of cell lines from the Cancer Cell Line Encyclopedia with predicted BRCA2 driver mutation status. Cell lines with mutations but of variance of unknown significance (VUS) were excluded from analysis. Cell lines with no mutation were considered ‘wildtype’. Driver annotations were based on OncoKB and Hotspots from cBioPortal. [file JCMM-29-e70845-s003.docx]

**Unmatched cell line collections are not optimal for identification of PARP inhibitor response and drug synergies**

Zoe Phan^1,2^, Kristine J. Fernandez^2^, C. Elizabeth Caldon^1,2*^

1. St Vincent’s Healthcare Clinical Campus, School of Clinical Medicine, UNSW Sydney, NSW, Australia
2. Garvan Institute of Medical Research, NSW, Australia

^*^ Correspondence: c.caldon@unsw.edu.au; [l.caldon@garvan.org.au](mailto:l.caldon@garvan.org.au)


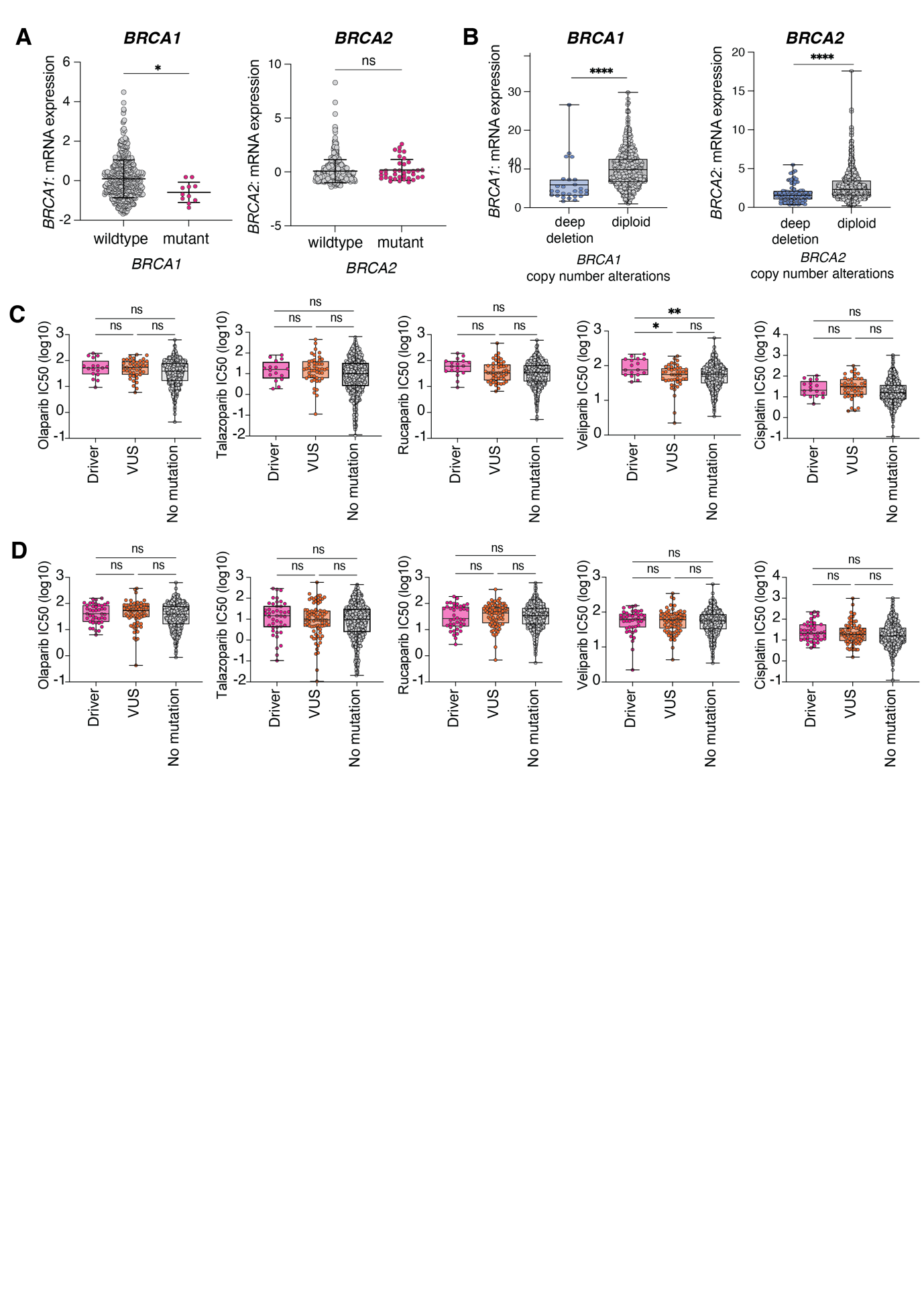
**Supplementary Figures**

**Supplementary Figure 1. BRCA1 and BRCA2 mRNA expression levels and IC_50_ response in cell lines with BRCA alterations.** **(A)** BRCA1 and BRCA2 mRNA expression in wildtype (n = 529 for BRCA1, n = 465 for BRCA2) and mutant (n = 11 for BRCA1, n = 37 for BRCA2) cell lines. **(B)** Cell lines with BRCA1 and BRCA2 deep deletions exhibit lower mRNA expression levels. BRCA1 expression is shown for deep deletion (n = 27) and diploid (n = 863) cell lines, while BRCA2 expression is shown for deep deletion (n = 84) and diploid (n = 818) cell lines. Statistical analyses were performed using an unpaired t-test. **(C)** IC_50_ response of various PARP inhibitors (olaparib, talazoparib, rucaparib, veliparib) and cisplatin in BRCA1-mutant cell lines with predicted driver mutations (n = 17-18), variance of unknown significance (VUS) (n = 43-48), and no documented mutations (n = 809-874). **(D)** IC_50_ response to the same treatments in BRCA2-mutant cell lines with predicted driver mutations (n = 40-43), VUS (n = 75-81), and no documented mutations (n = 752-819). Statistical analyses were performed using one-way ANOVA. *p < 0.01; **p < 0.001; **** p < 0.0001; ns = non-significant. Data were accessed via cBioPortal.


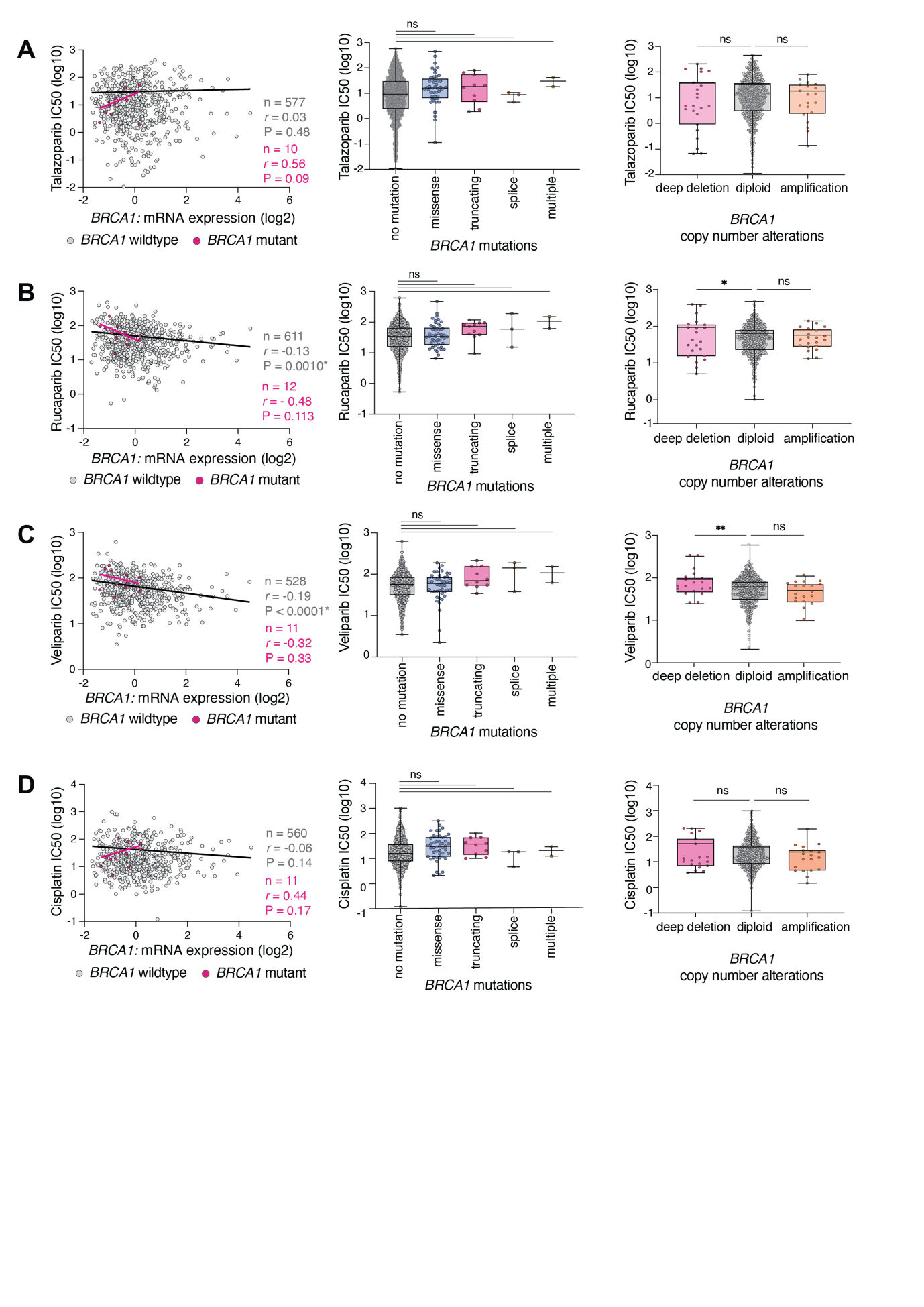


**Supplementary Figure 2. Altered BRCA1 expression is not associated with an increase in PARP inhibitor and platinum-based chemotherapy sensitivity in cell lines. (A-D)** IC_50_ responses to PARP inhibitors (olaparib, talazoparib, rucaparib, veliparib) and cisplatin in cell lines with BRCA1 alterations. Left panels: Correlation between IC_50_ response and BRCA1 expression relative to diploid samples. Red dots indicate cell lines with predicted driver mutations in BRCA1 (n = 10-12), while grey dots indicate BRCA1 wildtype (n = 528-611) cell lines. Best-fit linear regressions (red and black lines) illustrate positive or negative correlations. Middle panels: IC_50_ responses in BRCA1-mutant cell lines with different mutation types: no mutation (n = 809-872), missense (n = 46-50), truncating (n = 10-11), splice (n = 3) and multiple (n = 2). Right panels: IC_50_ response in cell lines with different BRCA1 copy number alteration statuses. IC_50_ values are shown for cell lines classified as deep deletion (n = 19-23), diploid (n = 517-563), and amplification (n = 19-21). Statistical analyses were determined by two-sided Pearson’s correlation test (left panel) and one-way ANOVA (middle and right panels). * p < 0.05; ** p < 0.01; ns = non-significant. Data were accessed via cBioPortal.


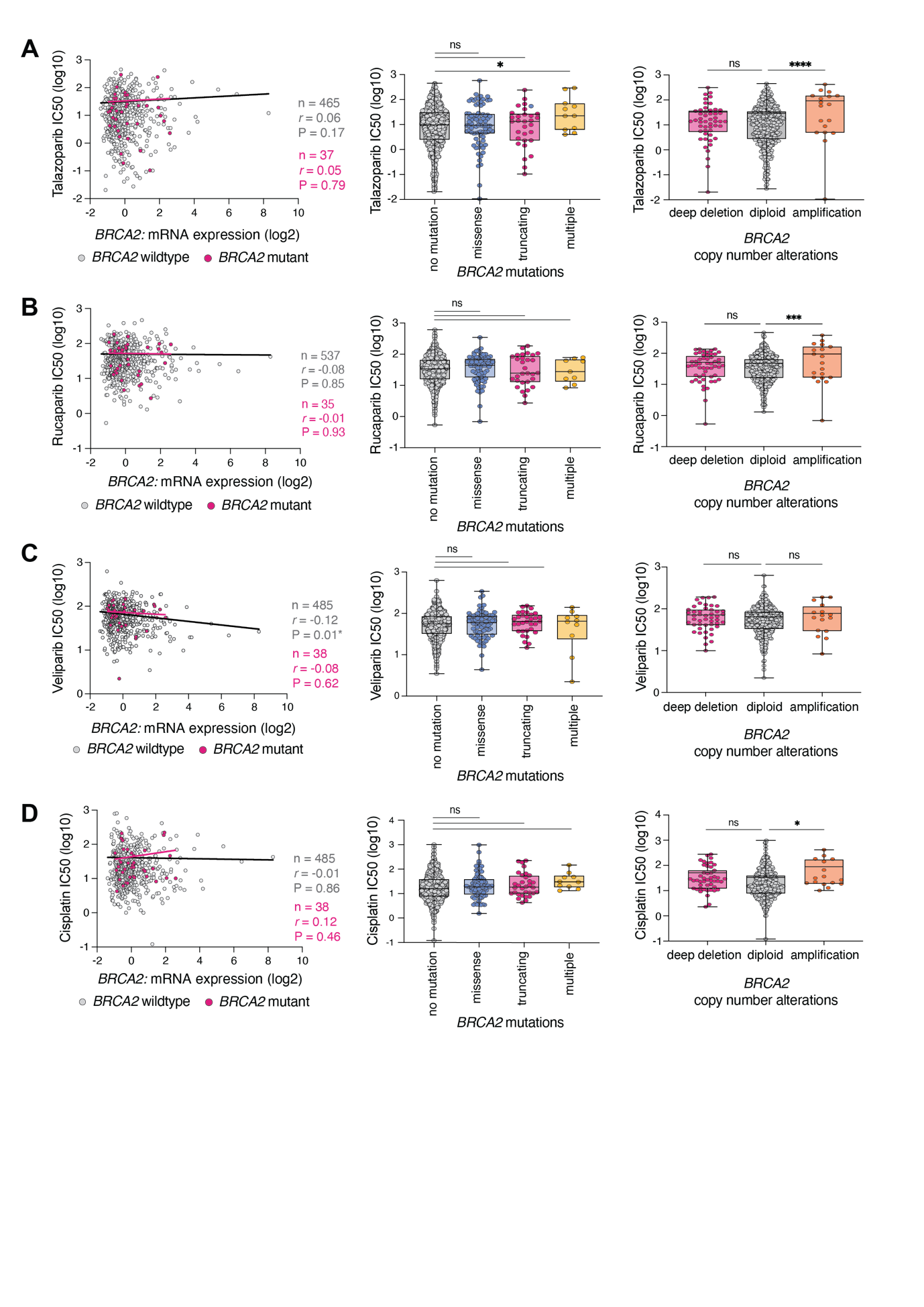


**Supplementary Figure 3. Altered BRCA2 expression is not associated with an increase in PARP inhibitor and platinum-based chemotherapy sensitivity in cell lines. (A-D)** IC_50_ responses to PARP inhibitors (olaparib, talazoparib, rucaparib, veliparib) and cisplatin in cell lines BRCA2 alterations. Left panels: Correlation between IC_50_ response and BRCA2 expression relative to diploid samples. Red dots indicate cell lines with predicted driver mutations in BRCA2 (n = 10-12), while grey dots indicate cell lines wildtype BRCA2 (n = 528-611) cell lines. Best-fit linear regressions (red and black lines) illustrate positive or negative correlations. Middle panels: IC_50_ responses in BRCA1-mutant cell lines with different mutation types: no mutation (n = 752-819), missense (n = 76-82), truncating (n = 30-32), and multiple (n = 9-10). Right panels: IC_50_ response in cell lines with different BRCA2 copy number alteration statuses. IC_50_ values are shown for cell lines classified as deep deletion (n = 47-53), diploid (n = 492-534), and amplification (n = 16-19). Statistical analyses were determined by two-sided Pearson’s correlation test (left panel) and one-way ANOVA (middle and right panels). * p < 0.05; *** p < 0.001; **** p < 0.0001; ns = non-significant. Data were accessed via cBioPortal.


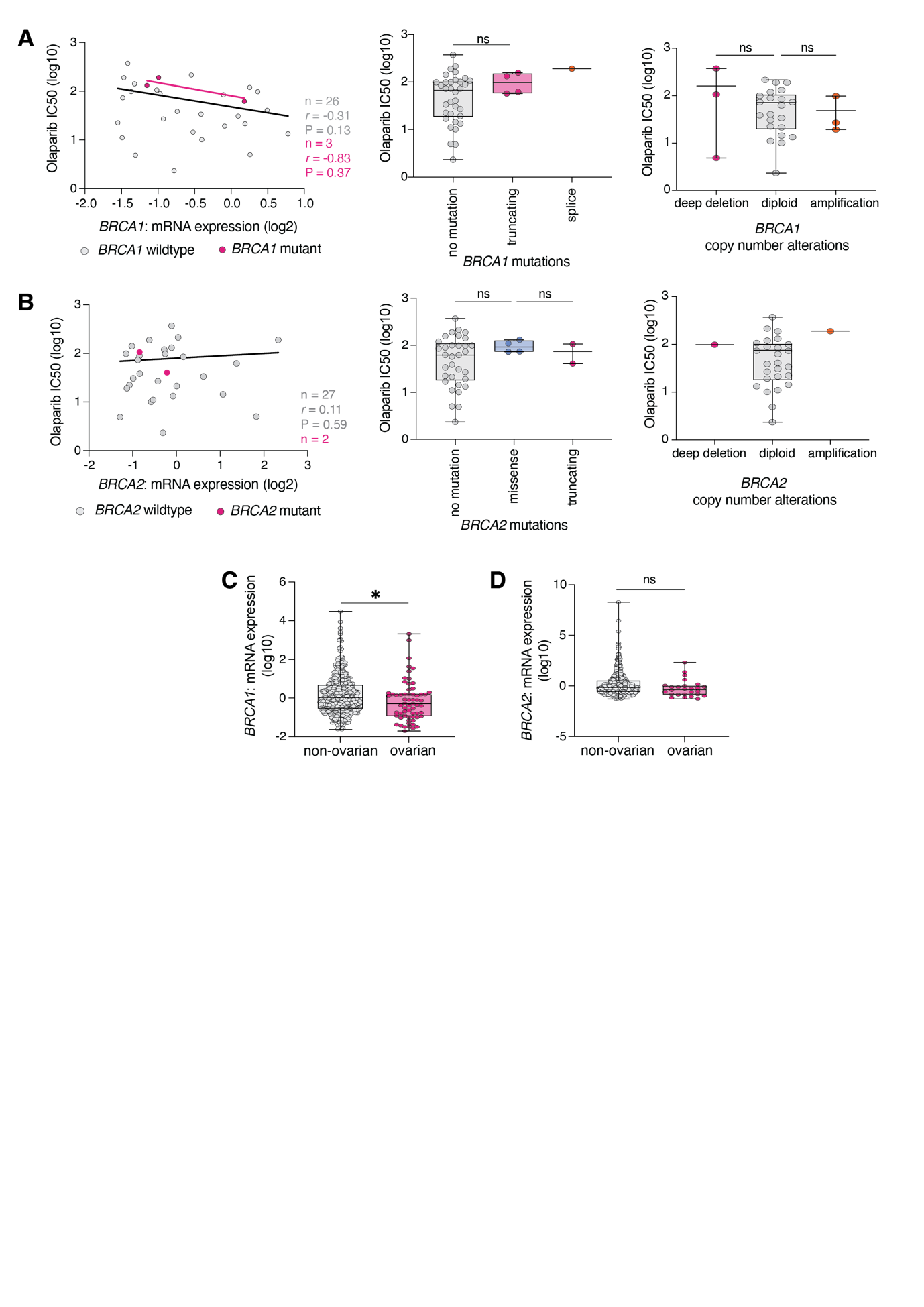
**Supplementary Figure 4. BRCA1 and BRCA2 altered ovarian cancer cell line collections are not associated with an increased sensitivity to a PARP inhibitor. (A-B)** IC_50_ responses to olaparib in ovarian cancer cell lines with **(A)** BRCA1 and **(B)** BRCA2 alterations. Left panels: Correlation between IC_50_ olaparib response and BRCA1/BRCA2 alterations relative to diploid samples. Red dots indicate cell lines with predicted driver mutations in BRCA1 (n = 3) and BRCA2 (n = 2), while grey dots indicate wildtype BRCA1 (n = 26) and BRCA2 (n = 27) cell lines. Best-fit linear regression (red and black lines) illustrates positive or negative correlations. Linear regression for BRCA2-mutant ovarian cancer cell lines were unable to be calculated with small sample size. Middle panels: Olaparib IC_50_ response in BRCA1- and BRCA2-mutant cell lines with different mutation types: no mutation (n = 34 for BRCA1, n = 33 for BRCA2), truncating (n = 4 for BRCA1, n = 2 for BRCA2), splice (n = 1 for BRCA1) and missense (n = 4 for BRCA2). Right panels: Olaparib IC_50_ response in ovarian cell lines with different BRCA1/2 copy number alteration statuses. IC_50_ values are shown for cell lines classified as deep deletion (n = 3 for BRCA1, n = 1 for BRCA2), diploid (n = 22 for BRCA1, n = 26 for BRCA2) and amplification (n = 3 for BRCA1, n = 1 for BRCA2). Comparisons for BRCA2 could not be calculated due to small sample size. Where statistical analyses could be determined, they were by two-sided Pearson’s correlation test (left panels) and one-way ANOVA (middle and right panels). **(C-D)** mRNA expression of **(C)** BRCA1 and **(D)** BRCA2 in ovarian (n = 63 for BRCA1, n = 26 for BRCA2) and non-ovarian cancer (n = 508 for BRCA1, n = 508 for BRCA2) cell lines. Statistical analyses were determined by unpaired t-test. * p < 0.05; ns = non-significant. Data were accessed via cBioPortal.
