# Supplementary material for: Unmatched Cell Line Collections Are Not Optimal for Identification of PARP Inhibitor Response and Drug Synergies
Source: J Cell Mol Med. 2025 Sep 22;29(18):e70845. doi: 10.1111/jcmm.70845 (PMC12451393; doi:10.1111/jcmm.70845)
Supplement: Supplementary file 2 — Figure S1: BRCA1 and BRCA2 mRNA expression levels and IC50 response in cell lines with BRCA alterations. (A) BRCA1 and BRCA2 mRNA expression in wildtype (n = 529 for BRCA1, n = 465 for BRCA2) and mutant (n = 11 for BRCA1, n = 37 for BRCA2) cell lines. (B) Cell lines with BRCA1 and BRCA2 deep deletions exhibit lower mRNA expression levels. BRCA1 expression is shown for deep deletion (n = 27) and diploid (n = 863) cell lines, while BRCA2 expression is shown for deep deletion (n = 84) and diploid (n = 818) cell lines. Statistical analyses were performed using an unpaired t‐test. (C) IC50 response of various PARP inhibitors (olaparib, talazoparib, rucaparib, veliparib) and cisplatin in BRCA1‐mutant cell lines with predicted driver mutations (n = 17–18), variance of unknown significance (VUS) (n = 43–48), and no documented mutations (n = 809–874). (D) IC50 response to the same treatments in BRCA2‐mutant cell lines with predicted driver mutations (n = 40–43), VUS (n = 75–81), and no documented mutations (n = 752–819). Statistical analyses were performed using one‐way ANOVA. *p < 0.01; **p < 0.001; **** p < 0.0001; ns = non‐significant. Data were accessed via cBioPortal. Figure S2: Altered BRCA1 expression is not associated with an increase in PARP inhibitor and platinum‐based chemotherapy sensitivity in cell lines. (A‐D) IC50 responses to PARP inhibitors (olaparib, talazoparib, rucaparib, veliparib) and cisplatin in cell lines with BRCA1 alterations. Left panels: Correlation between IC50 response and BRCA1 expression relative to diploid samples. Red dots indicate cell lines with predicted driver mutations in BRCA1 (n = 10–12), while grey dots indicate BRCA1 wildtype (n = 528–611) cell lines. Best‐fit linear regressions (red and black lines) illustrate positive or negative correlations. Middle panels: IC50 responses in BRCA1‐mutant cell lines with different mutation types: no mutation (n = 809–872), missense (n = 46–50), truncating (n = 10–11), splice (n = 3) and multiple [file JCMM-29-e70845-s002.docx]

**Unmatched cell line collections are not optimal for identification of PARP inhibitor response and drug synergies**

Zoe Phan^1,2^, Kristine J. Fernandez^2^, C. Elizabeth Caldon^1,2*^

1. St Vincent’s Healthcare Clinical Campus, School of Clinical Medicine, UNSW Sydney, NSW, Australia
2. Garvan Institute of Medical Research, NSW, Australia

^*^ Correspondence: c.caldon@unsw.edu.au; [l.caldon@garvan.org.au](mailto:l.caldon@garvan.org.au)

**Supplementary Methods**

1. **cBioPortal analysis**

Data on *BRCA1*, *BRCA2* and *PARP* mRNA expression relative to diploid samples, mutation type, copy number alteration status and fraction genome altered were extracted from cBioPortal using the Cancer Cell Line Encyclopedia (CCLE) (Broad, 2019) dataset (Data accessed on 20th February, 2024)^1-3^. Data was extracted alongside IC_50_ treatment response to olaparib, talazoparib, rucaparib, veliparib and cisplatin. The oncogenic effect of the variant was annotated using OncoKB™ and Hotspots. “Driver” and variance of unknown significance (“VUS”) mutations were annotated using OncoKB and Hotspots. Cell lines without gene profiling were excluded from the analysis. The list of cell lines along with *BRCA1* and *BRCA2* mutation status can be accessed in Supplementary Tables 1 and 2. To subset ovarian cancer specific cell lines, the search term “_OVARY” was used. Data was plotted and analysed using GraphPad Prism (v10.0).

1. **Genomics of Drug Sensitivity in Cancer analysis**

Data on the synergistic effects of combination olaparib and cisplatin were accessed from the Genomics of Drug Sensitivity in Cancer (GDSC) screening platform^4^. Combination olaparib and cisplatin was selected because platinum-based chemotherapy and PARP inhibitors have been investigated in over 80 clinical trials, and our previous findings showing *BRCA-*mutant derive greater benefit from PARP inhibitor-chemotherapy combinations compared to *BRCA-*wildtype patients^5^. Given that ovarian cancer cell lines were not included in this database, we investigated a collection of 46 breast cancer cell lines, as breast cancer is a malignancy where PARP inhibitors have also been approved. Synergy was determined using olaparib as the ‘anchor’ and cisplatin as the ‘library’ where the concentration of olaparib was 10 μM, and cisplatin contained a range doses, the max concentration being 4 μM. According to GDSC, synergy was determined as ΔEMax ≥ 0.2 and/or a ΔIC_50_ ≥ 3. Specifically, ΔIC_50_ is used to measure shifts in potency, calculated as the difference between the observed combination response and expected Bliss independence response (ΔIC_50_ = Bliss IC_50_ – combination IC_50_). ΔEMax indicates shifts in efficacy (ΔEMax = Bliss EMax – combination EMax). Where there were multiple values for a single cell line, the average was taken. Cell line *BRCA* mutation status and driver annotation was cross-referenced to the CCLE dataset. Cell lines where *BRCA* mutation status could not be determined were excluded from the analysis. All data were downloaded from GDSC Combinations, <https://gdsc-combinations.depmap.sanger.ac.uk/>.

1. **Cell culture**

*In vitro* experiments were performed using the isogenic ID8 models of ovarian cancer, (RRID: CVCL_IU14). Parental ID8 cells and CRISPR/Cas9-mutant cell lines, ID8*Trp53-/-*, ID8*Trp53-/-Brca1-/-* and ID8*Trp53-/-Brca2-/-* were kindly provided by Prof Iain McNeish (Imperial College London, UK)^6,7^. All cell lines were authenticated and was further confirmed by *Brca1* and *Brca2* Sanger sequencing. Note that a *Trp53* mutant background is utilized because *TP53* mutations are ubiquitous in ovarian cancer^8^. Cells were grown in DMEM (Gibco) media supplemented with 4% foetal bovine serum (Sigma-Aldrich HyClone), 1% penicillin/streptomycin (Invitrogen), and 1% insulin-transferrin selenium (Gibco). Cells were routinely passaged when they reached 80% confluency. To passage cells, first, cells were washed with warm phosphate-buffered saline (Gibco), and then incubated with 0.05% Trypsin/EDTA for 5 mins at 37 °C. Culturing media was used to deactivate the trypsin, and cells were transferred to a fresh tissue culture flask. Cells were maintained at 37 ˚C and 5% CO^­^_2_.

1. **Drugs**

For *in vitro* analyses, cell lines were treated with the following drugs or matched vehicle controls: olaparib (in DMSO; SelleckChem, S1060), carboplatin (in H_2_O; Abcam, ab120828) and paclitaxel (in DMSO; SelleckChem, S1150).

1. **Cell viability assay**

800 cells/well were plated into 96-well plates and media alone was used as a blank. After allowing cells to attach for 8 hours, cells were treated with increasing concentrations (10 pM – 100 μM) of olaparib. After 3 days, alamarBlue reagent (Thermo Fisher Scientific) was added in a 1:10 dilution, and after a 3-hour incubation at 37 °C, fluorescence (excitation 544 nm and emission 590 nm) was measured using the FLUROstar OPTIMA plate reader (BMG LabTech). To calculate cell viability curves, technical replicates were averaged, and the blank (media-only control) was subtracted from sample values. Data was then normalized to solvent control. These values were log transformed, normalized and plotted with a nonlinear fit of least square analysis. IC_50_ values were determined using GraphPad Prism (v10.0).

1. **Colony forming assay**

ID8 cells (300 cells/well) were seeded into 6-well plates as single-cells and left to attach for 6 hours. Cells were then treated with 1 μM olaparib, 6 μM carboplatin/8 nM paclitaxel or the combination of olaparib + carboplatin/paclitaxel and left for 7 days to allow colonies to grow. Media was refreshed once during the 7 days. The colonies were fixed with 16% trichloroacetic acid for 2 hours, washed with distilled water (dH_2_O), and air dried on the bench. Colonies were stained with 0.1% crystal violet for 1 hour before being washed with dH_2_O and left to air dry on the bench. Plates were scanned at 1200 dots per inch using the Perfection V800 Photo scanner (Epson Australia Pty Ltd). The colonies were quantified using ImageJ (v1.0)^9^ and the % area was calculated.

1. **Drug synergy assay**

Drug synergy assays were performed based on combination index (CI) method using CompuSyn software (v2.0) (Compusyn, INC). Cells were seeded at 800 cells/well in a 96-well plate and left to attach for 8 hours. Media alone was used as a blank. Based on the IC_50_ of each drug, six drug combinations (three above and three below the IC_50_) were tested to determine the dose-effect curve of olaparib and carboplatin/paclitaxel. Cell viability was measured after 3 days using a 1:10 dilution of alamarBlue and measured using the FLUROstar OPTIMA plate reader. The normalized values were inputted into the CompuSyn software to generate CI values. Microsoft Excel (v16.89.1) was then used to generate a three-colour scale based on the CI values obtained, where synergism (< 1) is represented by green, additive by yellow and antagonism (> 1) by red.

1. **Statistical analysis**

Pearson’s correlation coefficients were calculated using GraphPad Prism (v.10.0). To indicate negative or positive correlations, best-fit linear regressions were fitted onto the graph. All *in vitro* data are presented as the mean ± standard deviation unless otherwise specified. All *in vitro* experiments were performed in biological triplicates. Statistical significance was assessed by unpaired t-tests, two-sided t-test, one-way ANOVA, or two-way ANOVA followed by Tukey’s multiple comparisons test. The specific statistical analysis used for each experiment are provided in the figure legends. P-values < 0.05 were considered as statistically significant.

**Supplementary References**

1. Cerami, E.*, et al.* The cBio cancer genomics portal: an open platform for exploring multidimensional cancer genomics data. *Cancer Discov* **2**, 401-404 (2012).

2. Gao, J.*, et al.* Integrative analysis of complex cancer genomics and clinical profiles using the cBioPortal. *Sci Signal* **6**, pl1 (2013).

3. de Bruijn, I.*, et al.* Analysis and Visualization of Longitudinal Genomic and Clinical Data from the AACR Project GENIE Biopharma Collaborative in cBioPortal. *Cancer Res* **83**, 3861-3867 (2023).

4. Jaaks, P.*, et al.* Effective drug combinations in breast, colon and pancreatic cancer cells. *Nat* **603**, 166-173 (2022).

5. Phan, Z., Ford, C.E. & Caldon, C.E. DNA repair biomarkers to guide usage of combined PARP inhibitors and chemotherapy: A meta-analysis and systematic review. *Pharmacol Res* **196**, 106927 (2023).

6. Walton, J.B.*, et al.* CRISPR/Cas9-derived models of ovarian high grade serous carcinoma targeting Brca1, Pten and Nf1, and correlation with platinum sensitivity. *Sci Rep* **7**, 16827 (2017).

7. Walton, J.*, et al.* CRISPR/Cas9-Mediated Trp53 and Brca2 Knockout to Generate Improved Murine Models of Ovarian High-Grade Serous Carcinoma. *Cancer Res* **76**, 6118-6129 (2016).

8. Cancer Genome Atlas Research, N. Integrated genomic analyses of ovarian carcinoma. *Nat* **474**, 609-615 (2011).

9. Schneider, C.A., Rasband, W.S. & Eliceiri, K.W. NIH Image to ImageJ: 25 years of image analysis. *Nat Methods* **9**, 671-675 (2012).
