# Supplementary material for: Unmatched Cell Line Collections Are Not Optimal for Identification of PARP Inhibitor Response and Drug Synergies
Source: J Cell Mol Med. 2025 Sep 22;29(18):e70845. doi: 10.1111/jcmm.70845 (PMC12451393; doi:10.1111/jcmm.70845)
Supplement: Supplementary file 3 — Data S1: Supporting Information. [file JCMM-29-e70845-s001.docx]

**Supplementary Table 1**. List of cell lines from the Cancer Cell Line Encyclopedia with predicted *BRCA1* driver mutation status. Cell lines with mutations but of variance of unknown significance (VUS) were excluded from analysis. Cell lines with no mutation were considered ‘wildtype’. Driver annotations were based on OncoKB™ and Hotspots from cBioPortal.

| **Sample Id** | **BRCA1: Mutations** | **Type of mutation** | **Driver annotation** |
| --- | --- | --- | --- |
| HCC1395_BREAST | BRCA1: R1751* | Truncating | Driver |
| IGROV1_OVARY | BRCA1: K654Sfs*47 | Truncating | Driver |
| JHOS4_OVARY | BRCA1: X1760_splice | Splice | Driver |
| MDAMB436_BREAST | BRCA1: X1759_splice | Splice | Driver |
| ISHIKAWAHERAKLIO02ER_ENDOMETRIUM | BRCA1: X1803_splice | Splice | Driver |
| BHY_UPPER_AERODIGESTIVE_TRACT | BRCA1: G1709* | Truncating | Driver |
| SCLC21H_LUNG | BRCA1: V627Sfs*4 | Truncating | Driver |
| HT115_LARGE_INTESTINE | BRCA1: E577*, S763F, K1711R | Multiple | Driver |
| NCIH2347_LUNG | BRCA1: C1697F | Missense | Driver |
| DMS273_LUNG | BRCA1: R1699Q | Missense | Driver |
| JHOS2_OVARY | BRCA1: M1? | Truncating | Driver |
| HCC1954_BREAST | BRCA1: V1809Cfs*20 | Truncating | Driver |
| 8MGBA_CENTRAL_NERVOUS_SYSTEM | - | No mutation | No mutation |
| SUDHL8_HAEMATOPOIETIC_AND_LYMPHOID_TISSUE | - | No mutation | No mutation |
| EGI1_BILIARY_TRACT | - | No mutation | No mutation |
| BCPAP_THYROID | - | No mutation | No mutation |
| RH18_SOFT_TISSUE | - | No mutation | No mutation |
| MHHNB11_AUTONOMIC_GANGLIA | - | No mutation | No mutation |
| BICR22_UPPER_AERODIGESTIVE_TRACT | - | No mutation | No mutation |
| HCC2218_BREAST | - | No mutation | No mutation |
| OV56_OVARY | - | No mutation | No mutation |
| A3KAW_HAEMATOPOIETIC_AND_LYMPHOID_TISSUE | - | No mutation | No mutation |
| HS940T_FIBROBLAST | - | No mutation | No mutation |
| KYSE30_OESOPHAGUS | - | No mutation | No mutation |
| HS578T_BREAST | - | No mutation | No mutation |
| OVCAR8_OVARY | - | No mutation | No mutation |
| OVCAR4_OVARY | - | No mutation | No mutation |
| EFO21_OVARY | - | No mutation | No mutation |
| CAL851_BREAST | - | No mutation | No mutation |
| OVKATE_OVARY | - | No mutation | No mutation |
| JHH2_LIVER | - | No mutation | No mutation |
| HCC38_BREAST | - | No mutation | No mutation |
| RMGI_OVARY | - | No mutation | No mutation |
| SU8686_PANCREAS | - | No mutation | No mutation |
| BEN_LUNG | - | No mutation | No mutation |
| KYSE70_OESOPHAGUS | - | No mutation | No mutation |
| OV17R_OVARY | - | No mutation | No mutation |
| MDAMB134VI_BREAST | - | No mutation | No mutation |
| OVK18_OVARY | - | No mutation | No mutation |
| AU565_BREAST | - | No mutation | No mutation |
| ASPC1_PANCREAS | - | No mutation | No mutation |
| NCIH1915_LUNG | - | No mutation | No mutation |
| KLE_ENDOMETRIUM | - | No mutation | No mutation |
| CAKI1_KIDNEY | - | No mutation | No mutation |
| MCC13_SKIN | - | No mutation | No mutation |
| LP1_HAEMATOPOIETIC_AND_LYMPHOID_TISSUE | - | No mutation | No mutation |
| CL40_LARGE_INTESTINE | - | No mutation | No mutation |
| SNGM_ENDOMETRIUM | - | No mutation | No mutation |
| BHT101_THYROID | - | No mutation | No mutation |
| JIMT1_BREAST | - | No mutation | No mutation |
| 42MGBA_CENTRAL_NERVOUS_SYSTEM | - | No mutation | No mutation |
| HOP92_LUNG | - | No mutation | No mutation |
| ECGI10_OESOPHAGUS | - | No mutation | No mutation |
| MDAMB415_BREAST | - | No mutation | No mutation |
| HCC1419_BREAST | - | No mutation | No mutation |
| SKLMS1_SOFT_TISSUE | - | No mutation | No mutation |
| MOLP8_HAEMATOPOIETIC_AND_LYMPHOID_TISSUE | - | No mutation | No mutation |
| HSC2_UPPER_AERODIGESTIVE_TRACT | - | No mutation | No mutation |
| HPAFII_PANCREAS | - | No mutation | No mutation |
| KG1C_CENTRAL_NERVOUS_SYSTEM | - | No mutation | No mutation |
| SCC25_UPPER_AERODIGESTIVE_TRACT | - | No mutation | No mutation |
| NCIH1395_LUNG | - | No mutation | No mutation |
| NCIH596_LUNG | - | No mutation | No mutation |
| PC3_PROSTATE | - | No mutation | No mutation |
| SCC15_UPPER_AERODIGESTIVE_TRACT | - | No mutation | No mutation |
| UACC812_BREAST | - | No mutation | No mutation |
| MDAMB453_BREAST | - | No mutation | No mutation |
| HCC1187_BREAST | - | No mutation | No mutation |
| SKMEL24_SKIN | - | No mutation | No mutation |
| IALM_LUNG | - | No mutation | No mutation |
| ZR7530_BREAST | - | No mutation | No mutation |
| S117_SOFT_TISSUE | - | No mutation | No mutation |
| JVM2_HAEMATOPOIETIC_AND_LYMPHOID_TISSUE | - | No mutation | No mutation |
| KP3_PANCREAS | - | No mutation | No mutation |
| TC71_BONE | - | No mutation | No mutation |
| TE5_OESOPHAGUS | - | No mutation | No mutation |
| OUMS23_LARGE_INTESTINE | - | No mutation | No mutation |
| RPMI7951_SKIN | - | No mutation | No mutation |
| HCC44_LUNG | - | No mutation | No mutation |
| CAL120_BREAST | - | No mutation | No mutation |
| HCC56_LARGE_INTESTINE | - | No mutation | No mutation |
| A704_KIDNEY | - | No mutation | No mutation |
| KURAMOCHI_OVARY | - | No mutation | No mutation |
| MCC26_SKIN | - | No mutation | No mutation |
| TT_OESOPHAGUS | - | No mutation | No mutation |
| SIHA_CERVIX | - | No mutation | No mutation |
| SNU182_LIVER | - | No mutation | No mutation |
| OV90_OVARY | - | No mutation | No mutation |
| DETROIT562_UPPER_AERODIGESTIVE_TRACT | - | No mutation | No mutation |
| CAL54_KIDNEY | - | No mutation | No mutation |
| NCIH650_LUNG | - | No mutation | No mutation |
| SF268_CENTRAL_NERVOUS_SYSTEM | - | No mutation | No mutation |
| BICR31_UPPER_AERODIGESTIVE_TRACT | - | No mutation | No mutation |
| RCC10RGB_KIDNEY | - | No mutation | No mutation |
| MHHES1_BONE | - | No mutation | No mutation |
| EHEB_HAEMATOPOIETIC_AND_LYMPHOID_TISSUE | - | No mutation | No mutation |
| KALS1_CENTRAL_NERVOUS_SYSTEM | - | No mutation | No mutation |
| SCC4_UPPER_AERODIGESTIVE_TRACT | - | No mutation | No mutation |
| OVTOKO_OVARY | - | No mutation | No mutation |
| SNU475_LIVER | - | No mutation | No mutation |
| HCC1806_BREAST | - | No mutation | No mutation |
| 8305C_THYROID | - | No mutation | No mutation |
| SW1990_PANCREAS | - | No mutation | No mutation |
| EFE184_ENDOMETRIUM | - | No mutation | No mutation |
| ML1_THYROID | - | No mutation | No mutation |
| DBTRG05MG_CENTRAL_NERVOUS_SYSTEM | - | No mutation | No mutation |
| HUH7_LIVER | - | No mutation | No mutation |
| HPAC_PANCREAS | - | No mutation | No mutation |
| MDAMB175VII_BREAST | - | No mutation | No mutation |
| LXF289_LUNG | - | No mutation | No mutation |
| NCIH1793_LUNG | - | No mutation | No mutation |
| HUH1_LIVER | - | No mutation | No mutation |
| LS123_LARGE_INTESTINE | - | No mutation | No mutation |
| NCIH1693_LUNG | - | No mutation | No mutation |
| PC14_LUNG | - | No mutation | No mutation |
| SF539_CENTRAL_NERVOUS_SYSTEM | - | No mutation | No mutation |
| NCIH2444_LUNG | - | No mutation | No mutation |
| OAW28_OVARY | - | No mutation | No mutation |
| NCIH647_LUNG | - | No mutation | No mutation |
| EKVX_LUNG | - | No mutation | No mutation |
| HDQP1_BREAST | - | No mutation | No mutation |
| COLO783_SKIN | - | No mutation | No mutation |
| EFM192A_BREAST | - | No mutation | No mutation |
| FU97_STOMACH | - | No mutation | No mutation |
| TOV21G_OVARY | - | No mutation | No mutation |
| SCABER_URINARY_TRACT | - | No mutation | No mutation |
| NIHOVCAR3_OVARY | - | No mutation | No mutation |
| JHH1_LIVER | - | No mutation | No mutation |
| COLO680N_OESOPHAGUS | - | No mutation | No mutation |
| 647V_URINARY_TRACT | - | No mutation | No mutation |
| CFPAC1_PANCREAS | - | No mutation | No mutation |
| RERFLCMS_LUNG | - | No mutation | No mutation |
| CORL23_LUNG | - | No mutation | No mutation |
| CAL29_URINARY_TRACT | - | No mutation | No mutation |
| COLO792_SKIN | - | No mutation | No mutation |
| ES2_OVARY | - | No mutation | No mutation |
| EFM19_BREAST | - | No mutation | No mutation |
| BT483_BREAST | - | No mutation | No mutation |
| BICR78_UPPER_AERODIGESTIVE_TRACT | - | No mutation | No mutation |
| MCF7_BREAST | - | No mutation | No mutation |
| A673_BONE | - | No mutation | No mutation |
| SKMEL31_SKIN | - | No mutation | No mutation |
| U251MG_CENTRAL_NERVOUS_SYSTEM | - | No mutation | No mutation |
| OE21_OESOPHAGUS | - | No mutation | No mutation |
| HCC1569_BREAST | - | No mutation | No mutation |
| CAOV3_OVARY | - | No mutation | No mutation |
| MFE319_ENDOMETRIUM | - | No mutation | No mutation |
| NCIH716_LARGE_INTESTINE | - | No mutation | No mutation |
| SNU387_LIVER | - | No mutation | No mutation |
| NCIH2126_LUNG | - | No mutation | No mutation |
| CADOES1_BONE | - | No mutation | No mutation |
| NCIH3122_LUNG | - | No mutation | No mutation |
| WM115_SKIN | - | No mutation | No mutation |
| VMRCRCW_KIDNEY | - | No mutation | No mutation |
| OCIAML3_HAEMATOPOIETIC_AND_LYMPHOID_TISSUE | - | No mutation | No mutation |
| NCIH1792_LUNG | - | No mutation | No mutation |
| CORL105_LUNG | - | No mutation | No mutation |
| AGS_STOMACH | - | No mutation | No mutation |
| CAL78_BONE | - | No mutation | No mutation |
| OCILY19_HAEMATOPOIETIC_AND_LYMPHOID_TISSUE | - | No mutation | No mutation |
| DKMG_CENTRAL_NERVOUS_SYSTEM | - | No mutation | No mutation |
| HT55_LARGE_INTESTINE | - | No mutation | No mutation |
| PATU8988T_PANCREAS | - | No mutation | No mutation |
| UACC893_BREAST | - | No mutation | No mutation |
| OSRC2_KIDNEY | - | No mutation | No mutation |
| CASKI_CERVIX | - | No mutation | No mutation |
| HSC4_UPPER_AERODIGESTIVE_TRACT | - | No mutation | No mutation |
| TE10_OESOPHAGUS | - | No mutation | No mutation |
| TF1_HAEMATOPOIETIC_AND_LYMPHOID_TISSUE | - | No mutation | No mutation |
| MDST8_LARGE_INTESTINE | - | No mutation | No mutation |
| NCIH929_HAEMATOPOIETIC_AND_LYMPHOID_TISSUE | - | No mutation | No mutation |
| NCIH2196_LUNG | - | No mutation | No mutation |
| JVM3_HAEMATOPOIETIC_AND_LYMPHOID_TISSUE | - | No mutation | No mutation |
| MDAMB231_BREAST | - | No mutation | No mutation |
| OCUM1_STOMACH | - | No mutation | No mutation |
| SNB75_CENTRAL_NERVOUS_SYSTEM | - | No mutation | No mutation |
| BXPC3_PANCREAS | - | No mutation | No mutation |
| BT20_BREAST | - | No mutation | No mutation |
| HDMYZ_HAEMATOPOIETIC_AND_LYMPHOID_TISSUE | - | No mutation | No mutation |
| SKCO1_LARGE_INTESTINE | - | No mutation | No mutation |
| HEP3B217_LIVER | - | No mutation | No mutation |
| COLO668_LUNG | - | No mutation | No mutation |
| SW1710_URINARY_TRACT | - | No mutation | No mutation |
| RERFLCKJ_LUNG | - | No mutation | No mutation |
| SKHEP1_LIVER | - | No mutation | No mutation |
| ME1_HAEMATOPOIETIC_AND_LYMPHOID_TISSUE | - | No mutation | No mutation |
| MDAMB468_BREAST | - | No mutation | No mutation |
| SW982_SOFT_TISSUE | - | No mutation | No mutation |
| TT_THYROID | - | No mutation | No mutation |
| BFTC909_KIDNEY | - | No mutation | No mutation |
| NCIH2030_LUNG | - | No mutation | No mutation |
| CAOV4_OVARY | - | No mutation | No mutation |
| KMRC1_KIDNEY | - | No mutation | No mutation |
| DOTC24510_CERVIX | - | No mutation | No mutation |
| TE8_OESOPHAGUS | - | No mutation | No mutation |
| SNU1040_LARGE_INTESTINE | - | No mutation | No mutation |
| ECC12_STOMACH | - | No mutation | No mutation |
| TGBC11TKB_STOMACH | - | No mutation | No mutation |
| MIAPACA2_PANCREAS | - | No mutation | No mutation |
| NMCG1_CENTRAL_NERVOUS_SYSTEM | - | No mutation | No mutation |
| TYKNU_OVARY | - | No mutation | No mutation |
| NCIH1299_LUNG | - | No mutation | No mutation |
| NCIH2052_PLEURA | - | No mutation | No mutation |
| CALU6_LUNG | - | No mutation | No mutation |
| ABC1_LUNG | - | No mutation | No mutation |
| SAOS2_BONE | - | No mutation | No mutation |
| FTC133_THYROID | - | No mutation | No mutation |
| HT1197_URINARY_TRACT | - | No mutation | No mutation |
| RCM1_LARGE_INTESTINE | - | No mutation | No mutation |
| COLO684_ENDOMETRIUM | - | No mutation | No mutation |
| BT474_BREAST | - | No mutation | No mutation |
| NCIH2110_LUNG | - | No mutation | No mutation |
| KMH2_HAEMATOPOIETIC_AND_LYMPHOID_TISSUE | - | No mutation | No mutation |
| HCC202_BREAST | - | No mutation | No mutation |
| MELJUSO_SKIN | - | No mutation | No mutation |
| LOXIMVI_SKIN | - | No mutation | No mutation |
| MDAMB361_BREAST | - | No mutation | No mutation |
| HCC1143_BREAST | - | No mutation | No mutation |
| HT1080_SOFT_TISSUE | - | No mutation | No mutation |
| MESSA_SOFT_TISSUE | - | No mutation | No mutation |
| OE19_OESOPHAGUS | - | No mutation | No mutation |
| SNU398_LIVER | - | No mutation | No mutation |
| NCIH2228_LUNG | - | No mutation | No mutation |
| YH13_CENTRAL_NERVOUS_SYSTEM | - | No mutation | No mutation |
| COLO800_SKIN | - | No mutation | No mutation |
| SKES1_BONE | - | No mutation | No mutation |
| KYSE270_OESOPHAGUS | - | No mutation | No mutation |
| LNCAPCLONEFGC_PROSTATE | - | No mutation | No mutation |
| PSN1_PANCREAS | - | No mutation | No mutation |
| EFO27_OVARY | - | No mutation | No mutation |
| MPP89_PLEURA | - | No mutation | No mutation |
| NCIH1385_LUNG | - | No mutation | No mutation |
| OV7_OVARY | - | No mutation | No mutation |
| NUGC3_STOMACH | - | No mutation | No mutation |
| SW900_LUNG | - | No mutation | No mutation |
| HS766T_PANCREAS | - | No mutation | No mutation |
| ISTMES1_PLEURA | - | No mutation | No mutation |
| KYSE150_OESOPHAGUS | - | No mutation | No mutation |
| SJSA1_BONE | - | No mutation | No mutation |
| HT1376_URINARY_TRACT | - | No mutation | No mutation |
| RERFLCSQ1_LUNG | - | No mutation | No mutation |
| YKG1_CENTRAL_NERVOUS_SYSTEM | - | No mutation | No mutation |
| NCIH1573_LUNG | - | No mutation | No mutation |
| SKMES1_LUNG | - | No mutation | No mutation |
| G402_SOFT_TISSUE | - | No mutation | No mutation |
| SKMEL28_SKIN | - | No mutation | No mutation |
| NCIH2122_LUNG | - | No mutation | No mutation |
| NCIH2405_LUNG | - | No mutation | No mutation |
| CAL33_UPPER_AERODIGESTIVE_TRACT | - | No mutation | No mutation |
| DU4475_BREAST | - | No mutation | No mutation |
| RS411_HAEMATOPOIETIC_AND_LYMPHOID_TISSUE | - | No mutation | No mutation |
| NCIH1623_LUNG | - | No mutation | No mutation |
| CORL95_LUNG | - | No mutation | No mutation |
| NCIH292_LUNG | - | No mutation | No mutation |
| A101D_SKIN | - | No mutation | No mutation |
| ESO26_OESOPHAGUS | - | No mutation | No mutation |
| NCIH446_LUNG | - | No mutation | No mutation |
| NCIH226_LUNG | - | No mutation | No mutation |
| MEG01_HAEMATOPOIETIC_AND_LYMPHOID_TISSUE | - | No mutation | No mutation |
| HUCCT1_BILIARY_TRACT | - | No mutation | No mutation |
| NCIH2087_LUNG | - | No mutation | No mutation |
| TE11_OESOPHAGUS | - | No mutation | No mutation |
| GMS10_CENTRAL_NERVOUS_SYSTEM | - | No mutation | No mutation |
| 769P_KIDNEY | - | No mutation | No mutation |
| SBC5_LUNG | - | No mutation | No mutation |
| SNUC5_LARGE_INTESTINE | - | No mutation | No mutation |
| HOS_BONE | - | No mutation | No mutation |
| HT29_LARGE_INTESTINE | - | No mutation | No mutation |
| MFE280_ENDOMETRIUM | - | No mutation | No mutation |
| C32_SKIN | - | No mutation | No mutation |
| VMCUB1_URINARY_TRACT | - | No mutation | No mutation |
| FLO1_OESOPHAGUS | - | No mutation | No mutation |
| TE9_OESOPHAGUS | - | No mutation | No mutation |
| SCC9_UPPER_AERODIGESTIVE_TRACT | - | No mutation | No mutation |
| NCIH1437_LUNG | - | No mutation | No mutation |
| NCIH508_LARGE_INTESTINE | - | No mutation | No mutation |
| HCC827_LUNG | - | No mutation | No mutation |
| CAL148_BREAST | - | No mutation | No mutation |
| SET2_HAEMATOPOIETIC_AND_LYMPHOID_TISSUE | - | No mutation | No mutation |
| CL11_LARGE_INTESTINE | - | No mutation | No mutation |
| NCIH1666_LUNG | - | No mutation | No mutation |
| 2313287_STOMACH | - | No mutation | No mutation |
| WM793_SKIN | - | No mutation | No mutation |
| LS1034_LARGE_INTESTINE | - | No mutation | No mutation |
| HUTU80_SMALL_INTESTINE | - | No mutation | No mutation |
| SKUT1_SOFT_TISSUE | - | No mutation | No mutation |
| A498_KIDNEY | - | No mutation | No mutation |
| CAL27_UPPER_AERODIGESTIVE_TRACT | - | No mutation | No mutation |
| HL60_HAEMATOPOIETIC_AND_LYMPHOID_TISSUE | - | No mutation | No mutation |
| RERFGC1B_STOMACH | - | No mutation | No mutation |
| JAR_PLACENTA | - | No mutation | No mutation |
| NCIH196_LUNG | - | No mutation | No mutation |
| SIMA_AUTONOMIC_GANGLIA | - | No mutation | No mutation |
| SKNSH_AUTONOMIC_GANGLIA | - | No mutation | No mutation |
| HCC70_BREAST | - | No mutation | No mutation |
| NCIH3255_LUNG | - | No mutation | No mutation |
| A204_SOFT_TISSUE | - | No mutation | No mutation |
| KU1919_URINARY_TRACT | - | No mutation | No mutation |
| PANC0327_PANCREAS | - | No mutation | No mutation |
| LCLC103H_LUNG | - | No mutation | No mutation |
| SW1417_LARGE_INTESTINE | - | No mutation | No mutation |
| NCIH1944_LUNG | - | No mutation | No mutation |
| TE4_OESOPHAGUS | - | No mutation | No mutation |
| OACM51_OESOPHAGUS | - | No mutation | No mutation |
| MFE296_ENDOMETRIUM | - | No mutation | No mutation |
| TE441T_SOFT_TISSUE | - | No mutation | No mutation |
| C2BBE1_LARGE_INTESTINE | - | No mutation | No mutation |
| CHAGOK1_LUNG | - | No mutation | No mutation |
| NCIH1869_LUNG | - | No mutation | No mutation |
| COLO679_SKIN | - | No mutation | No mutation |
| CAL12T_LUNG | - | No mutation | No mutation |
| SKMEL30_SKIN | - | No mutation | No mutation |
| SHP77_LUNG | - | No mutation | No mutation |
| NCIH1781_LUNG | - | No mutation | No mutation |
| DV90_LUNG | - | No mutation | No mutation |
| SNU449_LIVER | - | No mutation | No mutation |
| DEL_HAEMATOPOIETIC_AND_LYMPHOID_TISSUE | - | No mutation | No mutation |
| SF126_CENTRAL_NERVOUS_SYSTEM | - | No mutation | No mutation |
| SW1116_LARGE_INTESTINE | - | No mutation | No mutation |
| NCIH1568_LUNG | - | No mutation | No mutation |
| FUOV1_OVARY | - | No mutation | No mutation |
| CALU3_LUNG | - | No mutation | No mutation |
| H4_CENTRAL_NERVOUS_SYSTEM | - | No mutation | No mutation |
| EOL1_HAEMATOPOIETIC_AND_LYMPHOID_TISSUE | - | No mutation | No mutation |
| SH4_SKIN | - | No mutation | No mutation |
| LOUCY_HAEMATOPOIETIC_AND_LYMPHOID_TISSUE | - | No mutation | No mutation |
| BV173_HAEMATOPOIETIC_AND_LYMPHOID_TISSUE | - | No mutation | No mutation |
| OVISE_OVARY | - | No mutation | No mutation |
| NCIH2291_LUNG | - | No mutation | No mutation |
| NCIH1355_LUNG | - | No mutation | No mutation |
| TE1_OESOPHAGUS | - | No mutation | No mutation |
| JHH6_LIVER | - | No mutation | No mutation |
| 8505C_THYROID | - | No mutation | No mutation |
| MEWO_SKIN | - | No mutation | No mutation |
| CL34_LARGE_INTESTINE | - | No mutation | No mutation |
| NCIH2029_LUNG | - | No mutation | No mutation |
| MDAMB157_BREAST | - | No mutation | No mutation |
| ONS76_CENTRAL_NERVOUS_SYSTEM | - | No mutation | No mutation |
| LU65_LUNG | - | No mutation | No mutation |
| HCC1428_BREAST | - | No mutation | No mutation |
| SNUC1_LARGE_INTESTINE | - | No mutation | No mutation |
| PANC0403_PANCREAS | - | No mutation | No mutation |
| SW620_LARGE_INTESTINE | - | No mutation | No mutation |
| IPC298_SKIN | - | No mutation | No mutation |
| KNS62_LUNG | - | No mutation | No mutation |
| NCIH520_LUNG | - | No mutation | No mutation |
| HS939T_SKIN | - | No mutation | No mutation |
| CW2_LARGE_INTESTINE | - | No mutation | No mutation |
| T24_URINARY_TRACT | - | No mutation | No mutation |
| NCIH1876_LUNG | - | No mutation | No mutation |
| NCIH2023_LUNG | - | No mutation | No mutation |
| 639V_URINARY_TRACT | - | No mutation | No mutation |
| NCIH841_LUNG | - | No mutation | No mutation |
| KYSE180_OESOPHAGUS | - | No mutation | No mutation |
| NOMO1_HAEMATOPOIETIC_AND_LYMPHOID_TISSUE | - | No mutation | No mutation |
| UACC257_SKIN | - | No mutation | No mutation |
| LOUNH91_LUNG | - | No mutation | No mutation |
| SW1463_LARGE_INTESTINE | - | No mutation | No mutation |
| CAL62_THYROID | - | No mutation | No mutation |
| CCFSTTG1_CENTRAL_NERVOUS_SYSTEM | - | No mutation | No mutation |
| NCIH1650_LUNG | - | No mutation | No mutation |
| NCIH1581_LUNG | - | No mutation | No mutation |
| PANC0203_PANCREAS | - | No mutation | No mutation |
| IGR1_SKIN | - | No mutation | No mutation |
| NCIH2085_LUNG | - | No mutation | No mutation |
| COLO741_SKIN | - | No mutation | No mutation |
| CAS1_CENTRAL_NERVOUS_SYSTEM | - | No mutation | No mutation |
| VCAP_PROSTATE | - | No mutation | No mutation |
| SKNAS_AUTONOMIC_GANGLIA | - | No mutation | No mutation |
| SW48_LARGE_INTESTINE | - | No mutation | No mutation |
| PANC0813_PANCREAS | - | No mutation | No mutation |
| OAW42_OVARY | - | No mutation | No mutation |
| ESS1_ENDOMETRIUM | - | No mutation | No mutation |
| BT549_BREAST | - | No mutation | No mutation |
| HT144_SKIN | - | No mutation | No mutation |
| HS683_CENTRAL_NERVOUS_SYSTEM | - | No mutation | No mutation |
| 786O_KIDNEY | - | No mutation | No mutation |
| HCC1500_BREAST | - | No mutation | No mutation |
| CHP126_AUTONOMIC_GANGLIA | - | No mutation | No mutation |
| KS1_CENTRAL_NERVOUS_SYSTEM | - | No mutation | No mutation |
| L1236_HAEMATOPOIETIC_AND_LYMPHOID_TISSUE | - | No mutation | No mutation |
| LN229_CENTRAL_NERVOUS_SYSTEM | - | No mutation | No mutation |
| CMLT1_HAEMATOPOIETIC_AND_LYMPHOID_TISSUE | - | No mutation | No mutation |
| KYM1_SOFT_TISSUE | - | No mutation | No mutation |
| SW1783_CENTRAL_NERVOUS_SYSTEM | - | No mutation | No mutation |
| MCAS_OVARY | - | No mutation | No mutation |
| SKMEL3_SKIN | - | No mutation | No mutation |
| NCIH1836_LUNG | - | No mutation | No mutation |
| SNU1_STOMACH | - | No mutation | No mutation |
| OPM2_HAEMATOPOIETIC_AND_LYMPHOID_TISSUE | - | No mutation | No mutation |
| RD_SOFT_TISSUE | - | No mutation | No mutation |
| TOV112D_OVARY | - | No mutation | No mutation |
| ECC10_STOMACH | - | No mutation | No mutation |
| NCIH358_LUNG | - | No mutation | No mutation |
| CAPAN2_PANCREAS | - | No mutation | No mutation |
| RT4_URINARY_TRACT | - | No mutation | No mutation |
| KU812_HAEMATOPOIETIC_AND_LYMPHOID_TISSUE | - | No mutation | No mutation |
| MOLM16_HAEMATOPOIETIC_AND_LYMPHOID_TISSUE | - | No mutation | No mutation |
| OCIAML5_HAEMATOPOIETIC_AND_LYMPHOID_TISSUE | - | No mutation | No mutation |
| TE6_OESOPHAGUS | - | No mutation | No mutation |
| HCC1599_BREAST | - | No mutation | No mutation |
| SKOV3_OVARY | - | No mutation | No mutation |
| NCIH810_LUNG | - | No mutation | No mutation |
| U2OS_BONE | - | No mutation | No mutation |
| C33A_CERVIX | - | No mutation | No mutation |
| PA1_OVARY | - | No mutation | No mutation |
| NCIH526_LUNG | - | No mutation | No mutation |
| KYSE450_OESOPHAGUS | - | No mutation | No mutation |
| RL952_ENDOMETRIUM | - | No mutation | No mutation |
| SUDHL4_HAEMATOPOIETIC_AND_LYMPHOID_TISSUE | - | No mutation | No mutation |
| OE33_OESOPHAGUS | - | No mutation | No mutation |
| U87MG_CENTRAL_NERVOUS_SYSTEM | - | No mutation | No mutation |
| MKN1_STOMACH | - | No mutation | No mutation |
| NCIH2170_LUNG | - | No mutation | No mutation |
| A427_LUNG | - | No mutation | No mutation |
| 5637_URINARY_TRACT | - | No mutation | No mutation |
| NCIH838_LUNG | - | No mutation | No mutation |
| HOP62_LUNG | - | No mutation | No mutation |
| SNU423_LIVER | - | No mutation | No mutation |
| T98G_CENTRAL_NERVOUS_SYSTEM | - | No mutation | No mutation |
| AN3CA_ENDOMETRIUM | - | No mutation | No mutation |
| NCIH1048_LUNG | - | No mutation | No mutation |
| HELA_CERVIX | - | No mutation | No mutation |
| MV411_HAEMATOPOIETIC_AND_LYMPHOID_TISSUE | - | No mutation | No mutation |
| NB4_HAEMATOPOIETIC_AND_LYMPHOID_TISSUE | - | No mutation | No mutation |
| NCIH82_LUNG | - | No mutation | No mutation |
| LS513_LARGE_INTESTINE | - | No mutation | No mutation |
| SW13_ADRENAL_CORTEX | - | No mutation | No mutation |
| DANG_PANCREAS | - | No mutation | No mutation |
| KMRC20_KIDNEY | - | No mutation | No mutation |
| SUPM2_HAEMATOPOIETIC_AND_LYMPHOID_TISSUE | - | No mutation | No mutation |
| PATU8902_PANCREAS | - | No mutation | No mutation |
| TCCSUP_URINARY_TRACT | - | No mutation | No mutation |
| SKMM2_HAEMATOPOIETIC_AND_LYMPHOID_TISSUE | - | No mutation | No mutation |
| NAMALWA_HAEMATOPOIETIC_AND_LYMPHOID_TISSUE | - | No mutation | No mutation |
| RKN_SOFT_TISSUE | - | No mutation | No mutation |
| IM95_STOMACH | - | No mutation | No mutation |
| DAOY_CENTRAL_NERVOUS_SYSTEM | - | No mutation | No mutation |
| NCIH2342_LUNG | - | No mutation | No mutation |
| SW626_OVARY | - | No mutation | No mutation |
| GCT_SOFT_TISSUE | - | No mutation | No mutation |
| A253_SALIVARY_GLAND | - | No mutation | No mutation |
| NCIH1092_LUNG | - | No mutation | No mutation |
| SNU61_LARGE_INTESTINE | - | No mutation | No mutation |
| EN_ENDOMETRIUM | - | No mutation | No mutation |
| SW1088_CENTRAL_NERVOUS_SYSTEM | - | No mutation | No mutation |
| HCT116_LARGE_INTESTINE | - | No mutation | No mutation |
| KO52_HAEMATOPOIETIC_AND_LYMPHOID_TISSUE | - | No mutation | No mutation |
| SKPNDW_BONE | - | No mutation | No mutation |
| EPLC272H_LUNG | - | No mutation | No mutation |
| PL21_HAEMATOPOIETIC_AND_LYMPHOID_TISSUE | - | No mutation | No mutation |
| CMK_HAEMATOPOIETIC_AND_LYMPHOID_TISSUE | - | No mutation | No mutation |
| NCIN87_STOMACH | - | No mutation | No mutation |
| NCIH2286_LUNG | - | No mutation | No mutation |
| SW403_LARGE_INTESTINE | - | No mutation | No mutation |
| NCIH2009_LUNG | - | No mutation | No mutation |
| MSTO211H_PLEURA | - | No mutation | No mutation |
| PECAPJ15_UPPER_AERODIGESTIVE_TRACT | - | No mutation | No mutation |
| HCC1937_BREAST | - | No mutation | No mutation |
| KPNRTBM1_AUTONOMIC_GANGLIA | - | No mutation | No mutation |
| KPNYN_AUTONOMIC_GANGLIA | - | No mutation | No mutation |
| KYSE520_OESOPHAGUS | - | No mutation | No mutation |
| DMS114_LUNG | - | No mutation | No mutation |
| CAMA1_BREAST | - | No mutation | No mutation |
| HEL_HAEMATOPOIETIC_AND_LYMPHOID_TISSUE | - | No mutation | No mutation |
| A4FUK_HAEMATOPOIETIC_AND_LYMPHOID_TISSUE | - | No mutation | No mutation |
| T84_LARGE_INTESTINE | - | No mutation | No mutation |
| NCIH522_LUNG | - | No mutation | No mutation |
| AM38_CENTRAL_NERVOUS_SYSTEM | - | No mutation | No mutation |
| MOLT16_HAEMATOPOIETIC_AND_LYMPHOID_TISSUE | - | No mutation | No mutation |
| KYSE410_OESOPHAGUS | - | No mutation | No mutation |
| SKMEL1_SKIN | - | No mutation | No mutation |
| NCIH510_LUNG | - | No mutation | No mutation |
| UMUC3_URINARY_TRACT | - | No mutation | No mutation |
| OC314_OVARY | - | No mutation | No mutation |
| TE15_OESOPHAGUS | - | No mutation | No mutation |
| KASUMI1_HAEMATOPOIETIC_AND_LYMPHOID_TISSUE | - | No mutation | No mutation |
| HGC27_STOMACH | - | No mutation | No mutation |
| MOLM13_HAEMATOPOIETIC_AND_LYMPHOID_TISSUE | - | No mutation | No mutation |
| HCC15_LUNG | - | No mutation | No mutation |
| NB1_AUTONOMIC_GANGLIA | - | No mutation | No mutation |
| NCIH1651_LUNG | - | No mutation | No mutation |
| NCIH460_LUNG | - | No mutation | No mutation |
| MELHO_SKIN | - | No mutation | No mutation |
| NCIH2081_LUNG | - | No mutation | No mutation |
| NCIH23_LUNG | - | No mutation | No mutation |
| RCHACV_HAEMATOPOIETIC_AND_LYMPHOID_TISSUE | - | No mutation | No mutation |
| KARPAS620_HAEMATOPOIETIC_AND_LYMPHOID_TISSUE | - | No mutation | No mutation |
| HUPT4_PANCREAS | - | No mutation | No mutation |
| KNS42_CENTRAL_NERVOUS_SYSTEM | - | No mutation | No mutation |
| CORL311_LUNG | - | No mutation | No mutation |
| RT112_URINARY_TRACT | - | No mutation | No mutation |
| KP2_PANCREAS | - | No mutation | No mutation |
| A375_SKIN | - | No mutation | No mutation |
| A2058_SKIN | - | No mutation | No mutation |
| GI1_CENTRAL_NERVOUS_SYSTEM | - | No mutation | No mutation |
| SIGM5_HAEMATOPOIETIC_AND_LYMPHOID_TISSUE | - | No mutation | No mutation |
| NCIH1435_LUNG | - | No mutation | No mutation |
| SKMEL5_SKIN | - | No mutation | No mutation |
| A172_CENTRAL_NERVOUS_SYSTEM | - | No mutation | No mutation |
| NCIH1734_LUNG | - | No mutation | No mutation |
| T47D_BREAST | - | No mutation | No mutation |
| CORL279_LUNG | - | No mutation | No mutation |
| RH41_SOFT_TISSUE | - | No mutation | No mutation |
| SKLU1_LUNG | - | No mutation | No mutation |
| LOVO_LARGE_INTESTINE | - | No mutation | No mutation |
| JURLMK1_HAEMATOPOIETIC_AND_LYMPHOID_TISSUE | - | No mutation | No mutation |
| HSC3_UPPER_AERODIGESTIVE_TRACT | - | No mutation | No mutation |
| QGP1_PANCREAS | - | No mutation | No mutation |
| NCIH1694_LUNG | - | No mutation | No mutation |
| COLO678_LARGE_INTESTINE | - | No mutation | No mutation |
| AMO1_HAEMATOPOIETIC_AND_LYMPHOID_TISSUE | - | No mutation | No mutation |
| HCC78_LUNG | - | No mutation | No mutation |
| SNU407_LARGE_INTESTINE | - | No mutation | No mutation |
| 697_HAEMATOPOIETIC_AND_LYMPHOID_TISSUE | - | No mutation | No mutation |
| NCIH1963_LUNG | - | No mutation | No mutation |
| NCIH2227_LUNG | - | No mutation | No mutation |
| U118MG_CENTRAL_NERVOUS_SYSTEM | - | No mutation | No mutation |
| D283MED_CENTRAL_NERVOUS_SYSTEM | - | No mutation | No mutation |
| NCIH1648_LUNG | - | No mutation | No mutation |
| LN18_CENTRAL_NERVOUS_SYSTEM | - | No mutation | No mutation |
| MM1S_HAEMATOPOIETIC_AND_LYMPHOID_TISSUE | - | No mutation | No mutation |
| A2780_OVARY | - | No mutation | No mutation |
| SW948_LARGE_INTESTINE | - | No mutation | No mutation |
| SNU16_STOMACH | - | No mutation | No mutation |
| NCIH146_LUNG | - | No mutation | No mutation |
| NCIH661_LUNG | - | No mutation | No mutation |
| ACHN_KIDNEY | - | No mutation | No mutation |
| HUPT3_PANCREAS | - | No mutation | No mutation |
| SW780_URINARY_TRACT | - | No mutation | No mutation |
| SNU5_STOMACH | - | No mutation | No mutation |
| NCIH209_LUNG | - | No mutation | No mutation |
| SISO_CERVIX | - | No mutation | No mutation |
| FADU_UPPER_AERODIGESTIVE_TRACT | - | No mutation | No mutation |
| SKNFI_AUTONOMIC_GANGLIA | - | No mutation | No mutation |
| NCIH2171_LUNG | - | No mutation | No mutation |
| SW756_CERVIX | - | No mutation | No mutation |
| KMS11_HAEMATOPOIETIC_AND_LYMPHOID_TISSUE | - | No mutation | No mutation |
| CAL51_BREAST | - | No mutation | No mutation |
| SKM1_HAEMATOPOIETIC_AND_LYMPHOID_TISSUE | - | No mutation | No mutation |
| MG63_BONE | - | No mutation | No mutation |
| KELLY_AUTONOMIC_GANGLIA | - | No mutation | No mutation |
| KYSE510_OESOPHAGUS | - | No mutation | No mutation |
| NCIH211_LUNG | - | No mutation | No mutation |
| KP4_PANCREAS | - | No mutation | No mutation |
| L428_HAEMATOPOIETIC_AND_LYMPHOID_TISSUE | - | No mutation | No mutation |
| NCIH1341_LUNG | - | No mutation | No mutation |
| HH_HAEMATOPOIETIC_AND_LYMPHOID_TISSUE | - | No mutation | No mutation |
| DMS53_LUNG | - | No mutation | No mutation |
| DOHH2_HAEMATOPOIETIC_AND_LYMPHOID_TISSUE | - | No mutation | No mutation |
| KE37_HAEMATOPOIETIC_AND_LYMPHOID_TISSUE | - | No mutation | No mutation |
| KOPN8_HAEMATOPOIETIC_AND_LYMPHOID_TISSUE | - | No mutation | No mutation |
| GAMG_CENTRAL_NERVOUS_SYSTEM | - | No mutation | No mutation |
| JHH4_LIVER | - | No mutation | No mutation |
| NCIH28_PLEURA | - | No mutation | No mutation |
| RDES_BONE | - | No mutation | No mutation |
| SUIT2_PANCREAS | - | No mutation | No mutation |
| GDM1_HAEMATOPOIETIC_AND_LYMPHOID_TISSUE | - | No mutation | No mutation |
| NCIH1975_LUNG | - | No mutation | No mutation |
| IGR37_SKIN | - | No mutation | No mutation |
| WSUDLCL2_HAEMATOPOIETIC_AND_LYMPHOID_TISSUE | - | No mutation | No mutation |
| ALLSIL_HAEMATOPOIETIC_AND_LYMPHOID_TISSUE | - | No mutation | No mutation |
| A549_LUNG | - | No mutation | No mutation |
| TALL1_HAEMATOPOIETIC_AND_LYMPHOID_TISSUE | - | No mutation | No mutation |
| DMS79_LUNG | - | No mutation | No mutation |
| KARPAS299_HAEMATOPOIETIC_AND_LYMPHOID_TISSUE | - | No mutation | No mutation |
| NCIH1755_LUNG | - | No mutation | No mutation |
| MS751_CERVIX | - | No mutation | No mutation |
| SW1573_LUNG | - | No mutation | No mutation |
| HDLM2_HAEMATOPOIETIC_AND_LYMPHOID_TISSUE | - | No mutation | No mutation |
| CALU1_LUNG | - | No mutation | No mutation |
| U266B1_HAEMATOPOIETIC_AND_LYMPHOID_TISSUE | - | No mutation | No mutation |
| LCLC97TM1_LUNG | - | No mutation | No mutation |
| SR786_HAEMATOPOIETIC_AND_LYMPHOID_TISSUE | - | No mutation | No mutation |
| P12ICHIKAWA_HAEMATOPOIETIC_AND_LYMPHOID_TISSUE | - | No mutation | No mutation |
| NCIH524_LUNG | - | No mutation | No mutation |
| GB1_CENTRAL_NERVOUS_SYSTEM | - | No mutation | No mutation |
| CAPAN1_PANCREAS | - | No mutation | No mutation |
| NCIH1618_LUNG | - | No mutation | No mutation |
| G401_SOFT_TISSUE | - | No mutation | No mutation |
| SNU175_LARGE_INTESTINE | - | No mutation | No mutation |
| DMS153_LUNG | - | No mutation | No mutation |
| LK2_LUNG | - | No mutation | No mutation |
| RPMI8402_HAEMATOPOIETIC_AND_LYMPHOID_TISSUE | - | No mutation | No mutation |
| GRANTA519_HAEMATOPOIETIC_AND_LYMPHOID_TISSUE | - | No mutation | No mutation |
| NUDUL1_HAEMATOPOIETIC_AND_LYMPHOID_TISSUE | - | No mutation | No mutation |
| G292CLONEA141B1_BONE | - | No mutation | No mutation |
| NCIH1155_LUNG | - | No mutation | No mutation |
| SKNEP1_BONE | - | No mutation | No mutation |
| RVH421_SKIN | - | No mutation | No mutation |
| CA46_HAEMATOPOIETIC_AND_LYMPHOID_TISSUE | - | No mutation | No mutation |
| RPMI8226_HAEMATOPOIETIC_AND_LYMPHOID_TISSUE | - | No mutation | No mutation |
| YAPC_PANCREAS | - | No mutation | No mutation |
| MEC1_HAEMATOPOIETIC_AND_LYMPHOID_TISSUE | - | No mutation | No mutation |
| PF382_HAEMATOPOIETIC_AND_LYMPHOID_TISSUE | - | No mutation | No mutation |
| SUDHL10_HAEMATOPOIETIC_AND_LYMPHOID_TISSUE | - | No mutation | No mutation |
| NCIH2452_PLEURA | - | No mutation | No mutation |
| LC1F_LUNG | - | No mutation | No mutation |
| MONOMAC6_HAEMATOPOIETIC_AND_LYMPHOID_TISSUE | - | No mutation | No mutation |
| GCIY_STOMACH | - | No mutation | No mutation |
| TT2609C02_THYROID | - | No mutation | No mutation |
| P31FUJ_HAEMATOPOIETIC_AND_LYMPHOID_TISSUE | - | No mutation | No mutation |
| SKGT4_OESOPHAGUS | - | No mutation | No mutation |
| SW837_LARGE_INTESTINE | - | No mutation | No mutation |
| NCIH1105_LUNG | - | No mutation | No mutation |
| KG1_HAEMATOPOIETIC_AND_LYMPHOID_TISSUE | - | No mutation | No mutation |
| OCIAML2_HAEMATOPOIETIC_AND_LYMPHOID_TISSUE | - | No mutation | No mutation |
| JJN3_HAEMATOPOIETIC_AND_LYMPHOID_TISSUE | - | No mutation | No mutation |
| RAJI_HAEMATOPOIETIC_AND_LYMPHOID_TISSUE | - | No mutation | No mutation |
| KMS12BM_HAEMATOPOIETIC_AND_LYMPHOID_TISSUE | - | No mutation | No mutation |
| HARA_LUNG | - | No mutation | No mutation |
| G361_SKIN | - | No mutation | No mutation |
| DAUDI_HAEMATOPOIETIC_AND_LYMPHOID_TISSUE | - | No mutation | No mutation |
| LAMA84_HAEMATOPOIETIC_AND_LYMPHOID_TISSUE | - | No mutation | No mutation |
| NCIH747_LARGE_INTESTINE | - | No mutation | No mutation |
| L540_HAEMATOPOIETIC_AND_LYMPHOID_TISSUE | - | No mutation | No mutation |
| NCIH441_LUNG | - | No mutation | No mutation |
| CORL88_LUNG | - | No mutation | No mutation |
| KARPAS422_HAEMATOPOIETIC_AND_LYMPHOID_TISSUE | - | No mutation | No mutation |
| L363_HAEMATOPOIETIC_AND_LYMPHOID_TISSUE | - | No mutation | No mutation |
| THP1_HAEMATOPOIETIC_AND_LYMPHOID_TISSUE | - | No mutation | No mutation |
| NCIH1436_LUNG | - | No mutation | No mutation |
| SUPB15_HAEMATOPOIETIC_AND_LYMPHOID_TISSUE | - | No mutation | No mutation |
| BL70_HAEMATOPOIETIC_AND_LYMPHOID_TISSUE | - | No mutation | No mutation |
| GA10_HAEMATOPOIETIC_AND_LYMPHOID_TISSUE | - | No mutation | No mutation |
| KATOIII_STOMACH | - | No mutation | No mutation |
| REH_HAEMATOPOIETIC_AND_LYMPHOID_TISSUE | - | No mutation | No mutation |
| SUDHL6_HAEMATOPOIETIC_AND_LYMPHOID_TISSUE | - | No mutation | No mutation |
| EBC1_LUNG | - | No mutation | No mutation |
| MHHCALL2_HAEMATOPOIETIC_AND_LYMPHOID_TISSUE | - | No mutation | No mutation |
| MOLT13_HAEMATOPOIETIC_AND_LYMPHOID_TISSUE | - | No mutation | No mutation |
| SF295_CENTRAL_NERVOUS_SYSTEM | - | No mutation | No mutation |
| MC116_HAEMATOPOIETIC_AND_LYMPHOID_TISSUE | - | No mutation | No mutation |
| NCIH69_LUNG | - | No mutation | No mutation |
| RL_HAEMATOPOIETIC_AND_LYMPHOID_TISSUE | - | No mutation | No mutation |
| HCC33_LUNG | - | No mutation | No mutation |
| BL41_HAEMATOPOIETIC_AND_LYMPHOID_TISSUE | - | No mutation | No mutation |
| DB_HAEMATOPOIETIC_AND_LYMPHOID_TISSUE | - | No mutation | No mutation |
| EJM_HAEMATOPOIETIC_AND_LYMPHOID_TISSUE | - | No mutation | No mutation |
| CHP212_AUTONOMIC_GANGLIA | - | No mutation | No mutation |
| NALM6_HAEMATOPOIETIC_AND_LYMPHOID_TISSUE | - | No mutation | No mutation |
| SKNDZ_AUTONOMIC_GANGLIA | - | No mutation | No mutation |
| JM1_HAEMATOPOIETIC_AND_LYMPHOID_TISSUE | - | No mutation | No mutation |
| NCIH1184_LUNG | - | No mutation | No mutation |
| P3HR1_HAEMATOPOIETIC_AND_LYMPHOID_TISSUE | - | No mutation | No mutation |
| K562_HAEMATOPOIETIC_AND_LYMPHOID_TISSUE | - | No mutation | No mutation |
| 22RV1_PROSTATE | - | No mutation | No mutation |
| ST486_HAEMATOPOIETIC_AND_LYMPHOID_TISSUE | - | No mutation | No mutation |
| TOLEDO_HAEMATOPOIETIC_AND_LYMPHOID_TISSUE | - | No mutation | No mutation |
| SUDHL5_HAEMATOPOIETIC_AND_LYMPHOID_TISSUE | - | No mutation | No mutation |
| EM2_HAEMATOPOIETIC_AND_LYMPHOID_TISSUE | - | No mutation | No mutation |
| SUDHL1_HAEMATOPOIETIC_AND_LYMPHOID_TISSUE | - | No mutation | No mutation |
| OCIM1_HAEMATOPOIETIC_AND_LYMPHOID_TISSUE | BRCA1: D1778G | Missense | VUS |
| GIMEN_AUTONOMIC_GANGLIA | BRCA1: R466Q | Missense | VUS |
| NCIH2066_LUNG | BRCA1: E1629V | Missense | VUS |
| HCC2157_BREAST | BRCA1: L30F | Missense | VUS |
| JEKO1_HAEMATOPOIETIC_AND_LYMPHOID_TISSUE | BRCA1: N742S | Missense | VUS |
| MKN7_STOMACH | BRCA1: V939L | Missense | VUS |
| NCIH1563_LUNG | BRCA1: A1453S | Missense | VUS |
| NCIH727_LUNG | BRCA1: W1815C | Missense | VUS |
| UO31_KIDNEY | BRCA1: K110N | Missense | VUS |
| UACC62_SKIN | BRCA1: P832S | Missense | VUS |
| LS180_LARGE_INTESTINE | BRCA1: N810T | Missense | VUS |
| NCIH2172_LUNG | BRCA1: Q1299H | Missense | VUS |
| BFTC905_URINARY_TRACT | BRCA1: D1398N | Missense | VUS |
| PLCPRF5_LIVER | BRCA1: I1026V | Missense | VUS |
| DND41_HAEMATOPOIETIC_AND_LYMPHOID_TISSUE | BRCA1: A1599V | Missense | VUS |
| NUGC4_STOMACH | BRCA1: I456T | Missense | VUS |
| KYSE140_OESOPHAGUS | BRCA1: I1058V | Missense | VUS |
| SNU81_LARGE_INTESTINE | BRCA1: E575K | Missense | VUS |
| JHH7_LIVER | BRCA1: L137I | Missense | VUS |
| HS746T_STOMACH | BRCA1: S186F | Missense | VUS |
| RKO_LARGE_INTESTINE | BRCA1: D435Y | Missense | VUS |
| KCL22_HAEMATOPOIETIC_AND_LYMPHOID_TISSUE | BRCA1: A1481V | Missense | VUS |
| VMRCRCZ_KIDNEY | BRCA1: I1044V | Missense | VUS |
| SW1271_LUNG | BRCA1: K1233I | Missense | VUS |
| NCIH1838_LUNG | BRCA1: C328Y | Missense | VUS |
| MKN45_STOMACH | BRCA1: S1577P | Missense | VUS |
| DU145_PROSTATE | BRCA1: E962K | Missense | VUS |
| NCIH1703_LUNG | BRCA1: G890V | Missense | VUS |
| HCT15_LARGE_INTESTINE | BRCA1: P1190H, S663N | Missense | VUS |
| KM12_LARGE_INTESTINE | BRCA1: P1579S | Missense | VUS |
| LS411N_LARGE_INTESTINE | BRCA1: L973F | Missense | VUS |
| CCK81_LARGE_INTESTINE | BRCA1: N976S, H553R, G401E | Missense | VUS |
| JURKAT_HAEMATOPOIETIC_AND_LYMPHOID_TISSUE | BRCA1: D1505N, C1270Y | Missense | VUS |
| PANC1005_PANCREAS | BRCA1: I571T | Missense | VUS |
| SUPT1_HAEMATOPOIETIC_AND_LYMPHOID_TISSUE | BRCA1: R1762K | Missense | VUS |
| HT_HAEMATOPOIETIC_AND_LYMPHOID_TISSUE | BRCA1: Q687P | Missense | VUS |

**Supplementary Table 2.** List of cell lines from the Cancer Cell Line Encyclopedia with predicted *BRCA2* driver mutation status. Cell lines with mutations but of variance of unknown significance (VUS) were excluded from analysis. Cell lines with no mutation were considered ‘wildtype’. Driver annotations were based on OncoKB™ and Hotspots from cBioPortal.

| **Sample Id** | **BRCA2: Mutations** | **Type of mutations** | **Driver annotation** |
| --- | --- | --- | --- |
| CAL851_BREAST | BRCA2: S1461Pfs*2 | Truncating | Driver |
| NCIH650_LUNG | BRCA2: S871* | Truncating | Driver |
| KURAMOCHI_OVARY | BRCA2: R2318* | Truncating | Driver |
| SNU1040_LARGE_INTESTINE | N/A | N/A | Driver |
| KCL22_HAEMATOPOIETIC_AND_LYMPHOID_TISSUE | BRCA2: N1287Kfs*2 | Truncating | Driver |
| DOTC24510_CERVIX | BRCA2: R3128* | Truncating | Driver |
| HCC202_BREAST | BRCA2: V1532Sfs*2 | Truncating | Driver |
| HT115_LARGE_INTESTINE | BRCA2: S2052*, E2258K, L29I, S3080Y | Multiple | Driver |
| HCC1569_BREAST | BRCA2: V1862*, N1100T | Multiple | Driver |
| BT474_BREAST | BRCA2: S3094* | Truncating | Driver |
| HCC1599_BREAST | BRCA2: K1517Ifs*23 | N/A | Driver |
| RL952_ENDOMETRIUM | BRCA2: Q1429Sfs*9, N1822Kfs*2, K2551E | Multiple | Driver |
| VMRCRCZ_KIDNEY | BRCA2: V726Sfs*25 | Truncating | Driver |
| CAPAN1_PANCREAS | BRCA2: V1532Sfs*2 | Truncating | Driver |
| SKMEL1_SKIN | BRCA2: E2981Rfs*37 | Truncating | Driver |
| SNU407_LARGE_INTESTINE | BRCA2: I605Yfs*9 | Truncating | Driver |
| CMK_HAEMATOPOIETIC_AND_LYMPHOID_TISSUE | BRCA2: R2318* | Truncating | Driver |
| SNU81_LARGE_INTESTINE | BRCA2: E1928*, S1597Y | Multiple | Driver |
| OC314_OVARY | BRCA2: N1784Kfs*3 | Truncating | Driver |
| EHEB_HAEMATOPOIETIC_AND_LYMPHOID_TISSUE | BRCA2: V1532Sfs*2 | Truncating | Driver |
| HUTU80_SMALL_INTESTINE | BRCA2: V1532Sfs*2 | Truncating | Driver |
| SNU1_STOMACH | BRCA2: S309Hfs*15 | Truncating | Driver |
| CW2_LARGE_INTESTINE | BRCA2: N1287Ifs*6, X637_splice, Q2934*, D1476G, V2014E | Multiple | Driver |
| COLO783_SKIN | BRCA2: V1532Sfs*2 | Truncating | Driver |
| COLO679_SKIN | BRCA2: V1532Sfs*2 | Truncating | Driver |
| RKO_LARGE_INTESTINE | BRCA2: N1784Tfs*7 | Truncating | Driver |
| RVH421_SKIN | BRCA2: V1532Sfs*2 | Truncating | Driver |
| EN_ENDOMETRIUM | BRCA2: E2981Rfs*37 | Truncating | Driver |
| SW48_LARGE_INTESTINE | BRCA2: K1691Nfs*15, N1784Tfs*7 | Truncating | Driver |
| TE9_OESOPHAGUS | BRCA2: S1989* | Truncating | Driver |
| SNU175_LARGE_INTESTINE | N/A | N/A | Driver |
| NCIH1693_LUNG | BRCA2: V1532Sfs*2 | Truncating | Driver |
| HCT116_LARGE_INTESTINE | BRCA2: I2675Dfs*6 | Truncating | Driver |
| FADU_UPPER_AERODIGESTIVE_TRACT | BRCA2: N863Kfs*18 | Truncating | Driver |
| HCT15_LARGE_INTESTINE | BRCA2: C1200*, N1784Tfs*7, R2784Q, A487V | Multiple | Driver |
| CAL51_BREAST | BRCA2: N986Ifs*5 | Truncating | Driver |
| CORL88_LUNG | BRCA2: E443* | Truncating | Driver |
| NCIH747_LARGE_INTESTINE | BRCA2: V1532Sfs*2 | Truncating | Driver |
| GRANTA519_HAEMATOPOIETIC_AND_LYMPHOID_TISSUE | BRCA2: W1692Mfs*3 | Truncating | Driver |
| KM12_LARGE_INTESTINE | BRCA2: N1784Tfs*7, K1565N | Multiple | Driver |
| KO52_HAEMATOPOIETIC_AND_LYMPHOID_TISSUE | N/A | N/A | Driver |
| HCC1395_BREAST | BRCA2: E1593* | Truncating | Driver |
| P3HR1_HAEMATOPOIETIC_AND_LYMPHOID_TISSUE | N/A | N/A | Driver |
| EGI1_BILIARY_TRACT | - | No mutation | No mutation |
| BCPAP_THYROID | N/A | N/A | No mutation |
| OVK18_OVARY | - | No mutation | No mutation |
| NCIH1563_LUNG | - | No mutation | No mutation |
| KYSE70_OESOPHAGUS | - | No mutation | No mutation |
| BICR22_UPPER_AERODIGESTIVE_TRACT | - | No mutation | No mutation |
| GIMEN_AUTONOMIC_GANGLIA | - | No mutation | No mutation |
| CAKI1_KIDNEY | - | No mutation | No mutation |
| KYSE30_OESOPHAGUS | N/A | N/A | No mutation |
| MOLP8_HAEMATOPOIETIC_AND_LYMPHOID_TISSUE | - | No mutation | No mutation |
| HCC2218_BREAST | - | No mutation | No mutation |
| RMGI_OVARY | - | No mutation | No mutation |
| HOP92_LUNG | - | No mutation | No mutation |
| OVISE_OVARY | - | No mutation | No mutation |
| ASPC1_PANCREAS | - | No mutation | No mutation |
| MHHES1_BONE | - | No mutation | No mutation |
| SKMEL24_SKIN | - | No mutation | No mutation |
| BV173_HAEMATOPOIETIC_AND_LYMPHOID_TISSUE | - | No mutation | No mutation |
| HS940T_FIBROBLAST | N/A | N/A | No mutation |
| BHT101_THYROID | - | No mutation | No mutation |
| OV56_OVARY | - | No mutation | No mutation |
| LP1_HAEMATOPOIETIC_AND_LYMPHOID_TISSUE | - | No mutation | No mutation |
| DKMG_CENTRAL_NERVOUS_SYSTEM | - | No mutation | No mutation |
| RH18_SOFT_TISSUE | - | No mutation | No mutation |
| BICR31_UPPER_AERODIGESTIVE_TRACT | N/A | N/A | No mutation |
| U251MG_CENTRAL_NERVOUS_SYSTEM | - | No mutation | No mutation |
| SCLC21H_LUNG | - | No mutation | No mutation |
| RS411_HAEMATOPOIETIC_AND_LYMPHOID_TISSUE | N/A | N/A | No mutation |
| KLE_ENDOMETRIUM | - | No mutation | No mutation |
| OV17R_OVARY | - | No mutation | No mutation |
| SCC15_UPPER_AERODIGESTIVE_TRACT | N/A | N/A | No mutation |
| NCIH2291_LUNG | - | No mutation | No mutation |
| HPAFII_PANCREAS | N/A | N/A | No mutation |
| MDAMB415_BREAST | - | No mutation | No mutation |
| YKG1_CENTRAL_NERVOUS_SYSTEM | - | No mutation | No mutation |
| OAW42_OVARY | - | No mutation | No mutation |
| SF268_CENTRAL_NERVOUS_SYSTEM | - | No mutation | No mutation |
| ML1_THYROID | N/A | N/A | No mutation |
| KMS12BM_HAEMATOPOIETIC_AND_LYMPHOID_TISSUE | - | No mutation | No mutation |
| CHAGOK1_LUNG | N/A | N/A | No mutation |
| BT483_BREAST | - | No mutation | No mutation |
| SNGM_ENDOMETRIUM | - | No mutation | No mutation |
| 8305C_THYROID | - | No mutation | No mutation |
| KG1C_CENTRAL_NERVOUS_SYSTEM | N/A | N/A | No mutation |
| SKHEP1_LIVER | - | No mutation | No mutation |
| CAL54_KIDNEY | N/A | N/A | No mutation |
| SNU449_LIVER | - | No mutation | No mutation |
| SW1710_URINARY_TRACT | - | No mutation | No mutation |
| SKMEL31_SKIN | - | No mutation | No mutation |
| CFPAC1_PANCREAS | - | No mutation | No mutation |
| NCIH1395_LUNG | - | No mutation | No mutation |
| UACC62_SKIN | - | No mutation | No mutation |
| OCIM1_HAEMATOPOIETIC_AND_LYMPHOID_TISSUE | - | No mutation | No mutation |
| S117_SOFT_TISSUE | - | No mutation | No mutation |
| VMRCRCW_KIDNEY | - | No mutation | No mutation |
| UACC812_BREAST | N/A | N/A | No mutation |
| DMS273_LUNG | - | No mutation | No mutation |
| CAL120_BREAST | - | No mutation | No mutation |
| MDAMB175VII_BREAST | - | No mutation | No mutation |
| BICR78_UPPER_AERODIGESTIVE_TRACT | - | No mutation | No mutation |
| MFE280_ENDOMETRIUM | - | No mutation | No mutation |
| KMRC1_KIDNEY | - | No mutation | No mutation |
| FTC133_THYROID | - | No mutation | No mutation |
| EKVX_LUNG | - | No mutation | No mutation |
| HS578T_BREAST | - | No mutation | No mutation |
| NCIH3255_LUNG | - | No mutation | No mutation |
| OV90_OVARY | - | No mutation | No mutation |
| A498_KIDNEY | - | No mutation | No mutation |
| LXF289_LUNG | - | No mutation | No mutation |
| ES2_OVARY | - | No mutation | No mutation |
| KS1_CENTRAL_NERVOUS_SYSTEM | - | No mutation | No mutation |
| RERFLCKJ_LUNG | - | No mutation | No mutation |
| HELA_CERVIX | - | No mutation | No mutation |
| NCIH226_LUNG | - | No mutation | No mutation |
| SIHA_CERVIX | - | No mutation | No mutation |
| JHH4_LIVER | - | No mutation | No mutation |
| HL60_HAEMATOPOIETIC_AND_LYMPHOID_TISSUE | - | No mutation | No mutation |
| KARPAS620_HAEMATOPOIETIC_AND_LYMPHOID_TISSUE | - | No mutation | No mutation |
| MDAMB134VI_BREAST | N/A | N/A | No mutation |
| SW1783_CENTRAL_NERVOUS_SYSTEM | - | No mutation | No mutation |
| GMS10_CENTRAL_NERVOUS_SYSTEM | - | No mutation | No mutation |
| HCC1428_BREAST | - | No mutation | No mutation |
| BEN_LUNG | N/A | N/A | No mutation |
| CL40_LARGE_INTESTINE | - | No mutation | No mutation |
| ONS76_CENTRAL_NERVOUS_SYSTEM | - | No mutation | No mutation |
| NCIH1581_LUNG | - | No mutation | No mutation |
| OCIAML3_HAEMATOPOIETIC_AND_LYMPHOID_TISSUE | - | No mutation | No mutation |
| PSN1_PANCREAS | - | No mutation | No mutation |
| ME1_HAEMATOPOIETIC_AND_LYMPHOID_TISSUE | - | No mutation | No mutation |
| ECGI10_OESOPHAGUS | - | No mutation | No mutation |
| NCIH1623_LUNG | - | No mutation | No mutation |
| DBTRG05MG_CENTRAL_NERVOUS_SYSTEM | - | No mutation | No mutation |
| HSC4_UPPER_AERODIGESTIVE_TRACT | - | No mutation | No mutation |
| TE10_OESOPHAGUS | N/A | N/A | No mutation |
| SW1417_LARGE_INTESTINE | - | No mutation | No mutation |
| PC3_PROSTATE | N/A | N/A | No mutation |
| COLO680N_OESOPHAGUS | - | No mutation | No mutation |
| JIMT1_BREAST | - | No mutation | No mutation |
| HDMYZ_HAEMATOPOIETIC_AND_LYMPHOID_TISSUE | - | No mutation | No mutation |
| A3KAW_HAEMATOPOIETIC_AND_LYMPHOID_TISSUE | - | No mutation | No mutation |
| PC14_LUNG | - | No mutation | No mutation |
| JHH6_LIVER | - | No mutation | No mutation |
| SCC9_UPPER_AERODIGESTIVE_TRACT | - | No mutation | No mutation |
| WM115_SKIN | - | No mutation | No mutation |
| LCLC103H_LUNG | - | No mutation | No mutation |
| CAL148_BREAST | N/A | N/A | No mutation |
| NMCG1_CENTRAL_NERVOUS_SYSTEM | - | No mutation | No mutation |
| NCIH2122_LUNG | - | No mutation | No mutation |
| 5637_URINARY_TRACT | - | No mutation | No mutation |
| 647V_URINARY_TRACT | - | No mutation | No mutation |
| ECC10_STOMACH | - | No mutation | No mutation |
| AU565_BREAST | - | No mutation | No mutation |
| NCIH1437_LUNG | - | No mutation | No mutation |
| SKLMS1_SOFT_TISSUE | - | No mutation | No mutation |
| SKCO1_LARGE_INTESTINE | N/A | N/A | No mutation |
| OPM2_HAEMATOPOIETIC_AND_LYMPHOID_TISSUE | - | No mutation | No mutation |
| NCIH596_LUNG | - | No mutation | No mutation |
| SW982_SOFT_TISSUE | - | No mutation | No mutation |
| CAL29_URINARY_TRACT | - | No mutation | No mutation |
| CASKI_CERVIX | - | No mutation | No mutation |
| MDST8_LARGE_INTESTINE | - | No mutation | No mutation |
| TT_THYROID | - | No mutation | No mutation |
| NCIH2030_LUNG | N/A | N/A | No mutation |
| 8505C_THYROID | - | No mutation | No mutation |
| EOL1_HAEMATOPOIETIC_AND_LYMPHOID_TISSUE | - | No mutation | No mutation |
| RPMI7951_SKIN | - | No mutation | No mutation |
| ECC12_STOMACH | N/A | N/A | No mutation |
| SU8686_PANCREAS | - | No mutation | No mutation |
| ISTMES1_PLEURA | N/A | N/A | No mutation |
| SH4_SKIN | - | No mutation | No mutation |
| HCC1419_BREAST | - | No mutation | No mutation |
| NCIH460_LUNG | - | No mutation | No mutation |
| NCIH2087_LUNG | - | No mutation | No mutation |
| MIAPACA2_PANCREAS | - | No mutation | No mutation |
| NCIH2444_LUNG | - | No mutation | No mutation |
| RERFLCMS_LUNG | - | No mutation | No mutation |
| SKUT1_SOFT_TISSUE | - | No mutation | No mutation |
| RPMI8226_HAEMATOPOIETIC_AND_LYMPHOID_TISSUE | - | No mutation | No mutation |
| EFE184_ENDOMETRIUM | N/A | N/A | No mutation |
| KMRC20_KIDNEY | - | No mutation | No mutation |
| TE11_OESOPHAGUS | - | No mutation | No mutation |
| NUGC4_STOMACH | N/A | N/A | No mutation |
| EFO21_OVARY | - | No mutation | No mutation |
| JURLMK1_HAEMATOPOIETIC_AND_LYMPHOID_TISSUE | - | No mutation | No mutation |
| SCABER_URINARY_TRACT | - | No mutation | No mutation |
| HCC78_LUNG | - | No mutation | No mutation |
| NCIH1792_LUNG | - | No mutation | No mutation |
| H4_CENTRAL_NERVOUS_SYSTEM | - | No mutation | No mutation |
| OAW28_OVARY | N/A | N/A | No mutation |
| U87MG_CENTRAL_NERVOUS_SYSTEM | - | No mutation | No mutation |
| BFTC905_URINARY_TRACT | - | No mutation | No mutation |
| CAL33_UPPER_AERODIGESTIVE_TRACT | - | No mutation | No mutation |
| VCAP_PROSTATE | - | No mutation | No mutation |
| SKMM2_HAEMATOPOIETIC_AND_LYMPHOID_TISSUE | - | No mutation | No mutation |
| CORL105_LUNG | N/A | N/A | No mutation |
| COLO684_ENDOMETRIUM | - | No mutation | No mutation |
| MDAMB157_BREAST | N/A | N/A | No mutation |
| A704_KIDNEY | - | No mutation | No mutation |
| OV7_OVARY | - | No mutation | No mutation |
| SNU475_LIVER | N/A | N/A | No mutation |
| NCIH1650_LUNG | N/A | N/A | No mutation |
| SCC25_UPPER_AERODIGESTIVE_TRACT | - | No mutation | No mutation |
| NCIH1573_LUNG | N/A | N/A | No mutation |
| HCC1500_BREAST | - | No mutation | No mutation |
| HUH1_LIVER | - | No mutation | No mutation |
| SNU387_LIVER | - | No mutation | No mutation |
| OE21_OESOPHAGUS | - | No mutation | No mutation |
| JHH1_LIVER | - | No mutation | No mutation |
| SKMEL30_SKIN | - | No mutation | No mutation |
| LNCAPCLONEFGC_PROSTATE | N/A | N/A | No mutation |
| YH13_CENTRAL_NERVOUS_SYSTEM | - | No mutation | No mutation |
| L428_HAEMATOPOIETIC_AND_LYMPHOID_TISSUE | - | No mutation | No mutation |
| MHHNB11_AUTONOMIC_GANGLIA | - | No mutation | No mutation |
| HCC44_LUNG | - | No mutation | No mutation |
| SW13_ADRENAL_CORTEX | - | No mutation | No mutation |
| 42MGBA_CENTRAL_NERVOUS_SYSTEM | - | No mutation | No mutation |
| A101D_SKIN | - | No mutation | No mutation |
| T47D_BREAST | - | No mutation | No mutation |
| OVCAR8_OVARY | - | No mutation | No mutation |
| CALU3_LUNG | - | No mutation | No mutation |
| ABC1_LUNG | - | No mutation | No mutation |
| MG63_BONE | - | No mutation | No mutation |
| CAS1_CENTRAL_NERVOUS_SYSTEM | - | No mutation | No mutation |
| CAL62_THYROID | - | No mutation | No mutation |
| UACC893_BREAST | - | No mutation | No mutation |
| IGR1_SKIN | - | No mutation | No mutation |
| HPAC_PANCREAS | N/A | N/A | No mutation |
| MV411_HAEMATOPOIETIC_AND_LYMPHOID_TISSUE | - | No mutation | No mutation |
| NCIH522_LUNG | N/A | N/A | No mutation |
| EJM_HAEMATOPOIETIC_AND_LYMPHOID_TISSUE | - | No mutation | No mutation |
| LOUNH91_LUNG | - | No mutation | No mutation |
| NCIH647_LUNG | - | No mutation | No mutation |
| KALS1_CENTRAL_NERVOUS_SYSTEM | N/A | N/A | No mutation |
| OCILY19_HAEMATOPOIETIC_AND_LYMPHOID_TISSUE | - | No mutation | No mutation |
| SF126_CENTRAL_NERVOUS_SYSTEM | - | No mutation | No mutation |
| TT_OESOPHAGUS | - | No mutation | No mutation |
| CHP126_AUTONOMIC_GANGLIA | - | No mutation | No mutation |
| HT55_LARGE_INTESTINE | - | No mutation | No mutation |
| CAL78_BONE | - | No mutation | No mutation |
| NCIH2172_LUNG | - | No mutation | No mutation |
| JVM3_HAEMATOPOIETIC_AND_LYMPHOID_TISSUE | - | No mutation | No mutation |
| NCIH1963_LUNG | - | No mutation | No mutation |
| HEL_HAEMATOPOIETIC_AND_LYMPHOID_TISSUE | N/A | N/A | No mutation |
| SNU423_LIVER | - | No mutation | No mutation |
| RERFLCSQ1_LUNG | - | No mutation | No mutation |
| LK2_LUNG | - | No mutation | No mutation |
| SW620_LARGE_INTESTINE | - | No mutation | No mutation |
| BFTC909_KIDNEY | N/A | N/A | No mutation |
| NCIH1975_LUNG | - | No mutation | No mutation |
| NCIH2228_LUNG | N/A | N/A | No mutation |
| NCIH82_LUNG | - | No mutation | No mutation |
| HCC2157_BREAST | - | No mutation | No mutation |
| OVTOKO_OVARY | - | No mutation | No mutation |
| NCIH2023_LUNG | - | No mutation | No mutation |
| MONOMAC6_HAEMATOPOIETIC_AND_LYMPHOID_TISSUE | - | No mutation | No mutation |
| SJSA1_BONE | - | No mutation | No mutation |
| KNS42_CENTRAL_NERVOUS_SYSTEM | - | No mutation | No mutation |
| AMO1_HAEMATOPOIETIC_AND_LYMPHOID_TISSUE | - | No mutation | No mutation |
| CALU1_LUNG | N/A | N/A | No mutation |
| NCIH2405_LUNG | - | No mutation | No mutation |
| MEG01_HAEMATOPOIETIC_AND_LYMPHOID_TISSUE | - | No mutation | No mutation |
| AM38_CENTRAL_NERVOUS_SYSTEM | - | No mutation | No mutation |
| NCIH2110_LUNG | - | No mutation | No mutation |
| SNB75_CENTRAL_NERVOUS_SYSTEM | - | No mutation | No mutation |
| LCLC97TM1_LUNG | - | No mutation | No mutation |
| UO31_KIDNEY | N/A | N/A | No mutation |
| KOPN8_HAEMATOPOIETIC_AND_LYMPHOID_TISSUE | - | No mutation | No mutation |
| 769P_KIDNEY | N/A | N/A | No mutation |
| PECAPJ15_UPPER_AERODIGESTIVE_TRACT | - | No mutation | No mutation |
| NCIH841_LUNG | - | No mutation | No mutation |
| LS180_LARGE_INTESTINE | - | No mutation | No mutation |
| RCHACV_HAEMATOPOIETIC_AND_LYMPHOID_TISSUE | - | No mutation | No mutation |
| KYSE270_OESOPHAGUS | N/A | N/A | No mutation |
| KYSE520_OESOPHAGUS | - | No mutation | No mutation |
| MELHO_SKIN | - | No mutation | No mutation |
| NCIH211_LUNG | - | No mutation | No mutation |
| OSRC2_KIDNEY | - | No mutation | No mutation |
| A172_CENTRAL_NERVOUS_SYSTEM | N/A | N/A | No mutation |
| TCCSUP_URINARY_TRACT | N/A | N/A | No mutation |
| NCIH1299_LUNG | N/A | N/A | No mutation |
| RD_SOFT_TISSUE | N/A | N/A | No mutation |
| RKN_SOFT_TISSUE | - | No mutation | No mutation |
| LC1F_LUNG | N/A | N/A | No mutation |
| GCT_SOFT_TISSUE | N/A | N/A | No mutation |
| NCIH2029_LUNG | - | No mutation | No mutation |
| NIHOVCAR3_OVARY | N/A | N/A | No mutation |
| HEP3B217_LIVER | - | No mutation | No mutation |
| GB1_CENTRAL_NERVOUS_SYSTEM | - | No mutation | No mutation |
| TOV21G_OVARY | - | No mutation | No mutation |
| BHY_UPPER_AERODIGESTIVE_TRACT | N/A | N/A | No mutation |
| NCIH2081_LUNG | - | No mutation | No mutation |
| NCIH2171_LUNG | N/A | N/A | No mutation |
| D283MED_CENTRAL_NERVOUS_SYSTEM | - | No mutation | No mutation |
| TE15_OESOPHAGUS | - | No mutation | No mutation |
| MESSA_SOFT_TISSUE | - | No mutation | No mutation |
| NCIH1048_LUNG | - | No mutation | No mutation |
| SKMES1_LUNG | - | No mutation | No mutation |
| TF1_HAEMATOPOIETIC_AND_LYMPHOID_TISSUE | N/A | N/A | No mutation |
| HDQP1_BREAST | - | No mutation | No mutation |
| LAMA84_HAEMATOPOIETIC_AND_LYMPHOID_TISSUE | - | No mutation | No mutation |
| DU4475_BREAST | - | No mutation | No mutation |
| SBC5_LUNG | - | No mutation | No mutation |
| CORL23_LUNG | - | No mutation | No mutation |
| MS751_CERVIX | - | No mutation | No mutation |
| SNU182_LIVER | - | No mutation | No mutation |
| HT1197_URINARY_TRACT | - | No mutation | No mutation |
| MDAMB436_BREAST | - | No mutation | No mutation |
| WM793_SKIN | - | No mutation | No mutation |
| SKOV3_OVARY | - | No mutation | No mutation |
| NCIH1734_LUNG | - | No mutation | No mutation |
| LOXIMVI_SKIN | N/A | N/A | No mutation |
| QGP1_PANCREAS | - | No mutation | No mutation |
| JVM2_HAEMATOPOIETIC_AND_LYMPHOID_TISSUE | - | No mutation | No mutation |
| SW626_OVARY | - | No mutation | No mutation |
| PANC1005_PANCREAS | N/A | N/A | No mutation |
| MDAMB453_BREAST | - | No mutation | No mutation |
| IPC298_SKIN | - | No mutation | No mutation |
| 8MGBA_CENTRAL_NERVOUS_SYSTEM | - | No mutation | No mutation |
| KYM1_SOFT_TISSUE | - | No mutation | No mutation |
| SF539_CENTRAL_NERVOUS_SYSTEM | - | No mutation | No mutation |
| SW756_CERVIX | - | No mutation | No mutation |
| NCIH2085_LUNG | N/A | N/A | No mutation |
| NCIH727_LUNG | - | No mutation | No mutation |
| NCIH526_LUNG | - | No mutation | No mutation |
| SF295_CENTRAL_NERVOUS_SYSTEM | - | No mutation | No mutation |
| NCIH2126_LUNG | - | No mutation | No mutation |
| HS939T_SKIN | - | No mutation | No mutation |
| KYSE180_OESOPHAGUS | N/A | N/A | No mutation |
| RCM1_LARGE_INTESTINE | - | No mutation | No mutation |
| GI1_CENTRAL_NERVOUS_SYSTEM | - | No mutation | No mutation |
| PANC0813_PANCREAS | - | No mutation | No mutation |
| SKNAS_AUTONOMIC_GANGLIA | - | No mutation | No mutation |
| HSC2_UPPER_AERODIGESTIVE_TRACT | - | No mutation | No mutation |
| HCC1806_BREAST | N/A | N/A | No mutation |
| HCC56_LARGE_INTESTINE | - | No mutation | No mutation |
| HS683_CENTRAL_NERVOUS_SYSTEM | - | No mutation | No mutation |
| TGBC11TKB_STOMACH | - | No mutation | No mutation |
| HT1080_SOFT_TISSUE | - | No mutation | No mutation |
| HCC15_LUNG | - | No mutation | No mutation |
| WSUDLCL2_HAEMATOPOIETIC_AND_LYMPHOID_TISSUE | - | No mutation | No mutation |
| HOP62_LUNG | - | No mutation | No mutation |
| UMUC3_URINARY_TRACT | - | No mutation | No mutation |
| KYSE450_OESOPHAGUS | - | No mutation | No mutation |
| TT2609C02_THYROID | - | No mutation | No mutation |
| HCC827_LUNG | - | No mutation | No mutation |
| A427_LUNG | - | No mutation | No mutation |
| MOLM16_HAEMATOPOIETIC_AND_LYMPHOID_TISSUE | - | No mutation | No mutation |
| NCIH1944_LUNG | - | No mutation | No mutation |
| NCIH1155_LUNG | - | No mutation | No mutation |
| KMS11_HAEMATOPOIETIC_AND_LYMPHOID_TISSUE | - | No mutation | No mutation |
| K562_HAEMATOPOIETIC_AND_LYMPHOID_TISSUE | - | No mutation | No mutation |
| KMH2_HAEMATOPOIETIC_AND_LYMPHOID_TISSUE | - | No mutation | No mutation |
| NCIH2347_LUNG | - | No mutation | No mutation |
| NCIH2286_LUNG | - | No mutation | No mutation |
| KP4_PANCREAS | N/A | N/A | No mutation |
| KNS62_LUNG | - | No mutation | No mutation |
| MCF7_BREAST | - | No mutation | No mutation |
| YAPC_PANCREAS | - | No mutation | No mutation |
| A375_SKIN | - | No mutation | No mutation |
| SKGT4_OESOPHAGUS | - | No mutation | No mutation |
| SKMEL28_SKIN | N/A | N/A | No mutation |
| JJN3_HAEMATOPOIETIC_AND_LYMPHOID_TISSUE | - | No mutation | No mutation |
| G402_SOFT_TISSUE | - | No mutation | No mutation |
| CORL95_LUNG | - | No mutation | No mutation |
| LN229_CENTRAL_NERVOUS_SYSTEM | - | No mutation | No mutation |
| KP3_PANCREAS | - | No mutation | No mutation |
| OVKATE_OVARY | - | No mutation | No mutation |
| A4FUK_HAEMATOPOIETIC_AND_LYMPHOID_TISSUE | - | No mutation | No mutation |
| PLCPRF5_LIVER | N/A | N/A | No mutation |
| EFM192A_BREAST | - | No mutation | No mutation |
| NCIH1651_LUNG | - | No mutation | No mutation |
| CCFSTTG1_CENTRAL_NERVOUS_SYSTEM | - | No mutation | No mutation |
| A2780_OVARY | - | No mutation | No mutation |
| NCIH1781_LUNG | - | No mutation | No mutation |
| PANC0203_PANCREAS | - | No mutation | No mutation |
| BT549_BREAST | - | No mutation | No mutation |
| MDAMB231_BREAST | - | No mutation | No mutation |
| NCIH1385_LUNG | N/A | N/A | No mutation |
| VMCUB1_URINARY_TRACT | N/A | N/A | No mutation |
| MSTO211H_PLEURA | - | No mutation | No mutation |
| NCIH508_LARGE_INTESTINE | - | No mutation | No mutation |
| MKN1_STOMACH | N/A | N/A | No mutation |
| MCAS_OVARY | N/A | N/A | No mutation |
| THP1_HAEMATOPOIETIC_AND_LYMPHOID_TISSUE | - | No mutation | No mutation |
| KU1919_URINARY_TRACT | - | No mutation | No mutation |
| KYSE150_OESOPHAGUS | - | No mutation | No mutation |
| T24_URINARY_TRACT | N/A | N/A | No mutation |
| SUDHL4_HAEMATOPOIETIC_AND_LYMPHOID_TISSUE | - | No mutation | No mutation |
| NCIH292_LUNG | - | No mutation | No mutation |
| NCIH2009_LUNG | - | No mutation | No mutation |
| HT144_SKIN | N/A | N/A | No mutation |
| OACM51_OESOPHAGUS | - | No mutation | No mutation |
| NCIH1618_LUNG | N/A | N/A | No mutation |
| MCC26_SKIN | - | No mutation | No mutation |
| MPP89_PLEURA | - | No mutation | No mutation |
| TE441T_SOFT_TISSUE | N/A | N/A | No mutation |
| NB1_AUTONOMIC_GANGLIA | - | No mutation | No mutation |
| TOV112D_OVARY | - | No mutation | No mutation |
| PANC0327_PANCREAS | - | No mutation | No mutation |
| HUPT4_PANCREAS | - | No mutation | No mutation |
| A549_LUNG | - | No mutation | No mutation |
| U118MG_CENTRAL_NERVOUS_SYSTEM | - | No mutation | No mutation |
| CORL279_LUNG | - | No mutation | No mutation |
| T98G_CENTRAL_NERVOUS_SYSTEM | - | No mutation | No mutation |
| CAOV4_OVARY | - | No mutation | No mutation |
| NCIH1092_LUNG | - | No mutation | No mutation |
| DMS79_LUNG | - | No mutation | No mutation |
| TE5_OESOPHAGUS | - | No mutation | No mutation |
| DV90_LUNG | N/A | N/A | No mutation |
| TE8_OESOPHAGUS | - | No mutation | No mutation |
| NCIH2342_LUNG | - | No mutation | No mutation |
| SUDHL8_HAEMATOPOIETIC_AND_LYMPHOID_TISSUE | N/A | N/A | No mutation |
| SW900_LUNG | N/A | N/A | No mutation |
| NUGC3_STOMACH | N/A | N/A | No mutation |
| OUMS23_LARGE_INTESTINE | N/A | N/A | No mutation |
| JHH7_LIVER | - | No mutation | No mutation |
| KYSE140_OESOPHAGUS | - | No mutation | No mutation |
| A253_SALIVARY_GLAND | - | No mutation | No mutation |
| BXPC3_PANCREAS | - | No mutation | No mutation |
| C32_SKIN | - | No mutation | No mutation |
| CAL12T_LUNG | - | No mutation | No mutation |
| SCC4_UPPER_AERODIGESTIVE_TRACT | - | No mutation | No mutation |
| HS746T_STOMACH | - | No mutation | No mutation |
| EM2_HAEMATOPOIETIC_AND_LYMPHOID_TISSUE | - | No mutation | No mutation |
| MOLT16_HAEMATOPOIETIC_AND_LYMPHOID_TISSUE | - | No mutation | No mutation |
| A2058_SKIN | - | No mutation | No mutation |
| SUDHL10_HAEMATOPOIETIC_AND_LYMPHOID_TISSUE | N/A | N/A | No mutation |
| NCIH446_LUNG | - | No mutation | No mutation |
| L540_HAEMATOPOIETIC_AND_LYMPHOID_TISSUE | - | No mutation | No mutation |
| NCIH1793_LUNG | N/A | N/A | No mutation |
| NCIH1105_LUNG | - | No mutation | No mutation |
| T84_LARGE_INTESTINE | - | No mutation | No mutation |
| ESO26_OESOPHAGUS | - | No mutation | No mutation |
| FUOV1_OVARY | - | No mutation | No mutation |
| MEWO_SKIN | - | No mutation | No mutation |
| KP2_PANCREAS | - | No mutation | No mutation |
| NCIH1666_LUNG | - | No mutation | No mutation |
| SKMEL5_SKIN | - | No mutation | No mutation |
| CAMA1_BREAST | N/A | N/A | No mutation |
| A204_SOFT_TISSUE | N/A | N/A | No mutation |
| KU812_HAEMATOPOIETIC_AND_LYMPHOID_TISSUE | - | No mutation | No mutation |
| NCIH520_LUNG | - | No mutation | No mutation |
| HUH7_LIVER | - | No mutation | No mutation |
| CAL27_UPPER_AERODIGESTIVE_TRACT | - | No mutation | No mutation |
| SNU61_LARGE_INTESTINE | - | No mutation | No mutation |
| NCIH358_LUNG | - | No mutation | No mutation |
| SKNSH_AUTONOMIC_GANGLIA | - | No mutation | No mutation |
| RT4_URINARY_TRACT | - | No mutation | No mutation |
| SUIT2_PANCREAS | - | No mutation | No mutation |
| NCIH1703_LUNG | - | No mutation | No mutation |
| NCIH510_LUNG | - | No mutation | No mutation |
| SKLU1_LUNG | N/A | N/A | No mutation |
| KARPAS422_HAEMATOPOIETIC_AND_LYMPHOID_TISSUE | - | No mutation | No mutation |
| 639V_URINARY_TRACT | - | No mutation | No mutation |
| MEC1_HAEMATOPOIETIC_AND_LYMPHOID_TISSUE | - | No mutation | No mutation |
| AN3CA_ENDOMETRIUM | - | No mutation | No mutation |
| MKN7_STOMACH | - | No mutation | No mutation |
| KELLY_AUTONOMIC_GANGLIA | - | No mutation | No mutation |
| KASUMI1_HAEMATOPOIETIC_AND_LYMPHOID_TISSUE | - | No mutation | No mutation |
| RDES_BONE | N/A | N/A | No mutation |
| IM95_STOMACH | N/A | N/A | No mutation |
| NCIH69_LUNG | - | No mutation | No mutation |
| NCIH146_LUNG | - | No mutation | No mutation |
| LU65_LUNG | - | No mutation | No mutation |
| NAMALWA_HAEMATOPOIETIC_AND_LYMPHOID_TISSUE | - | No mutation | No mutation |
| SW1990_PANCREAS | - | No mutation | No mutation |
| SHP77_LUNG | - | No mutation | No mutation |
| NCIH2196_LUNG | N/A | N/A | No mutation |
| NCIH1755_LUNG | - | No mutation | No mutation |
| MM1S_HAEMATOPOIETIC_AND_LYMPHOID_TISSUE | - | No mutation | No mutation |
| U266B1_HAEMATOPOIETIC_AND_LYMPHOID_TISSUE | - | No mutation | No mutation |
| NCIH196_LUNG | N/A | N/A | No mutation |
| NCIH3122_LUNG | - | No mutation | No mutation |
| SET2_HAEMATOPOIETIC_AND_LYMPHOID_TISSUE | - | No mutation | No mutation |
| HCC1954_BREAST | - | No mutation | No mutation |
| CHP212_AUTONOMIC_GANGLIA | - | No mutation | No mutation |
| NCIH2227_LUNG | - | No mutation | No mutation |
| RAJI_HAEMATOPOIETIC_AND_LYMPHOID_TISSUE | - | No mutation | No mutation |
| RL_HAEMATOPOIETIC_AND_LYMPHOID_TISSUE | - | No mutation | No mutation |
| TE1_OESOPHAGUS | - | No mutation | No mutation |
| HARA_LUNG | - | No mutation | No mutation |
| KPNYN_AUTONOMIC_GANGLIA | - | No mutation | No mutation |
| NCIN87_STOMACH | - | No mutation | No mutation |
| HCC1187_BREAST | - | No mutation | No mutation |
| DAOY_CENTRAL_NERVOUS_SYSTEM | - | No mutation | No mutation |
| MKN45_STOMACH | - | No mutation | No mutation |
| HS766T_PANCREAS | - | No mutation | No mutation |
| NCIH2170_LUNG | N/A | N/A | No mutation |
| LS123_LARGE_INTESTINE | - | No mutation | No mutation |
| 2313287_STOMACH | - | No mutation | No mutation |
| SUPM2_HAEMATOPOIETIC_AND_LYMPHOID_TISSUE | - | No mutation | No mutation |
| DETROIT562_UPPER_AERODIGESTIVE_TRACT | - | No mutation | No mutation |
| HOS_BONE | - | No mutation | No mutation |
| OE19_OESOPHAGUS | - | No mutation | No mutation |
| NCIH1435_LUNG | - | No mutation | No mutation |
| HCC1143_BREAST | - | No mutation | No mutation |
| SW1088_CENTRAL_NERVOUS_SYSTEM | N/A | N/A | No mutation |
| SW1463_LARGE_INTESTINE | N/A | N/A | No mutation |
| DANG_PANCREAS | - | No mutation | No mutation |
| NCIH2052_PLEURA | - | No mutation | No mutation |
| NCIH1436_LUNG | - | No mutation | No mutation |
| CL11_LARGE_INTESTINE | - | No mutation | No mutation |
| SKPNDW_BONE | - | No mutation | No mutation |
| SW837_LARGE_INTESTINE | N/A | N/A | No mutation |
| 786O_KIDNEY | N/A | N/A | No mutation |
| HCC33_LUNG | N/A | N/A | No mutation |
| NCIH524_LUNG | - | No mutation | No mutation |
| NCIH441_LUNG | - | No mutation | No mutation |
| CAPAN2_PANCREAS | - | No mutation | No mutation |
| TC71_BONE | N/A | N/A | No mutation |
| PANC0403_PANCREAS | - | No mutation | No mutation |
| NCIH810_LUNG | - | No mutation | No mutation |
| IGR37_SKIN | - | No mutation | No mutation |
| CMLT1_HAEMATOPOIETIC_AND_LYMPHOID_TISSUE | - | No mutation | No mutation |
| G292CLONEA141B1_BONE | - | No mutation | No mutation |
| EFO27_OVARY | - | No mutation | No mutation |
| FU97_STOMACH | - | No mutation | No mutation |
| RPMI8402_HAEMATOPOIETIC_AND_LYMPHOID_TISSUE | - | No mutation | No mutation |
| SNU16_STOMACH | - | No mutation | No mutation |
| TE6_OESOPHAGUS | - | No mutation | No mutation |
| PATU8902_PANCREAS | - | No mutation | No mutation |
| NCIH1836_LUNG | - | No mutation | No mutation |
| LOVO_LARGE_INTESTINE | - | No mutation | No mutation |
| KPNRTBM1_AUTONOMIC_GANGLIA | N/A | N/A | No mutation |
| ISHIKAWAHERAKLIO02ER_ENDOMETRIUM | - | No mutation | No mutation |
| BL70_HAEMATOPOIETIC_AND_LYMPHOID_TISSUE | N/A | N/A | No mutation |
| NCIH1694_LUNG | - | No mutation | No mutation |
| EPLC272H_LUNG | - | No mutation | No mutation |
| ACHN_KIDNEY | - | No mutation | No mutation |
| SW780_URINARY_TRACT | - | No mutation | No mutation |
| NCIH1876_LUNG | - | No mutation | No mutation |
| HCC70_BREAST | - | No mutation | No mutation |
| OCIAML5_HAEMATOPOIETIC_AND_LYMPHOID_TISSUE | - | No mutation | No mutation |
| SKM1_HAEMATOPOIETIC_AND_LYMPHOID_TISSUE | N/A | N/A | No mutation |
| G361_SKIN | - | No mutation | No mutation |
| MC116_HAEMATOPOIETIC_AND_LYMPHOID_TISSUE | - | No mutation | No mutation |
| LS1034_LARGE_INTESTINE | - | No mutation | No mutation |
| HSC3_UPPER_AERODIGESTIVE_TRACT | N/A | N/A | No mutation |
| DMS114_LUNG | - | No mutation | No mutation |
| EFM19_BREAST | - | No mutation | No mutation |
| HT1376_URINARY_TRACT | - | No mutation | No mutation |
| NCIH23_LUNG | - | No mutation | No mutation |
| 697_HAEMATOPOIETIC_AND_LYMPHOID_TISSUE | - | No mutation | No mutation |
| PATU8988T_PANCREAS | - | No mutation | No mutation |
| A673_BONE | N/A | N/A | No mutation |
| EBC1_LUNG | - | No mutation | No mutation |
| CORL311_LUNG | N/A | N/A | No mutation |
| SIGM5_HAEMATOPOIETIC_AND_LYMPHOID_TISSUE | N/A | N/A | No mutation |
| DEL_HAEMATOPOIETIC_AND_LYMPHOID_TISSUE | - | No mutation | No mutation |
| NCIH2066_LUNG | N/A | N/A | No mutation |
| KARPAS299_HAEMATOPOIETIC_AND_LYMPHOID_TISSUE | - | No mutation | No mutation |
| GCIY_STOMACH | - | No mutation | No mutation |
| NCIH838_LUNG | - | No mutation | No mutation |
| NCIH1568_LUNG | - | No mutation | No mutation |
| RT112_URINARY_TRACT | N/A | N/A | No mutation |
| SW1271_LUNG | - | No mutation | No mutation |
| SKNFI_AUTONOMIC_GANGLIA | - | No mutation | No mutation |
| NCIH2452_PLEURA | - | No mutation | No mutation |
| RH41_SOFT_TISSUE | - | No mutation | No mutation |
| TYKNU_OVARY | - | No mutation | No mutation |
| BT20_BREAST | - | No mutation | No mutation |
| HCC1937_BREAST | - | No mutation | No mutation |
| SAOS2_BONE | - | No mutation | No mutation |
| G401_SOFT_TISSUE | - | No mutation | No mutation |
| U2OS_BONE | - | No mutation | No mutation |
| SKMEL3_SKIN | - | No mutation | No mutation |
| NCIH209_LUNG | - | No mutation | No mutation |
| SUDHL1_HAEMATOPOIETIC_AND_LYMPHOID_TISSUE | N/A | N/A | No mutation |
| NCIH1184_LUNG | N/A | N/A | No mutation |
| ESS1_ENDOMETRIUM | - | No mutation | No mutation |
| KG1_HAEMATOPOIETIC_AND_LYMPHOID_TISSUE | N/A | N/A | No mutation |
| NB4_HAEMATOPOIETIC_AND_LYMPHOID_TISSUE | - | No mutation | No mutation |
| OE33_OESOPHAGUS | - | No mutation | No mutation |
| SKNDZ_AUTONOMIC_GANGLIA | - | No mutation | No mutation |
| ALLSIL_HAEMATOPOIETIC_AND_LYMPHOID_TISSUE | - | No mutation | No mutation |
| L363_HAEMATOPOIETIC_AND_LYMPHOID_TISSUE | - | No mutation | No mutation |
| COLO678_LARGE_INTESTINE | - | No mutation | No mutation |
| JHOS2_OVARY | - | No mutation | No mutation |
| CAOV3_OVARY | N/A | N/A | No mutation |
| SW1573_LUNG | - | No mutation | No mutation |
| OCUM1_STOMACH | - | No mutation | No mutation |
| SW1116_LARGE_INTESTINE | N/A | N/A | No mutation |
| JEKO1_HAEMATOPOIETIC_AND_LYMPHOID_TISSUE | - | No mutation | No mutation |
| SKNEP1_BONE | - | No mutation | No mutation |
| DB_HAEMATOPOIETIC_AND_LYMPHOID_TISSUE | - | No mutation | No mutation |
| HT29_LARGE_INTESTINE | - | No mutation | No mutation |
| SKES1_BONE | - | No mutation | No mutation |
| NCIH716_LARGE_INTESTINE | - | No mutation | No mutation |
| LS513_LARGE_INTESTINE | - | No mutation | No mutation |
| NOMO1_HAEMATOPOIETIC_AND_LYMPHOID_TISSUE | - | No mutation | No mutation |
| GAMG_CENTRAL_NERVOUS_SYSTEM | N/A | N/A | No mutation |
| OCIAML2_HAEMATOPOIETIC_AND_LYMPHOID_TISSUE | - | No mutation | No mutation |
| SNUC1_LARGE_INTESTINE | - | No mutation | No mutation |
| AGS_STOMACH | - | No mutation | No mutation |
| PA1_OVARY | - | No mutation | No mutation |
| REH_HAEMATOPOIETIC_AND_LYMPHOID_TISSUE | - | No mutation | No mutation |
| NCIH1838_LUNG | - | No mutation | No mutation |
| KYSE410_OESOPHAGUS | - | No mutation | No mutation |
| CA46_HAEMATOPOIETIC_AND_LYMPHOID_TISSUE | - | No mutation | No mutation |
| SR786_HAEMATOPOIETIC_AND_LYMPHOID_TISSUE | - | No mutation | No mutation |
| SUDHL6_HAEMATOPOIETIC_AND_LYMPHOID_TISSUE | - | No mutation | No mutation |
| L1236_HAEMATOPOIETIC_AND_LYMPHOID_TISSUE | - | No mutation | No mutation |
| NUDUL1_HAEMATOPOIETIC_AND_LYMPHOID_TISSUE | - | No mutation | No mutation |
| COLO668_LUNG | - | No mutation | No mutation |
| LOUCY_HAEMATOPOIETIC_AND_LYMPHOID_TISSUE | - | No mutation | No mutation |
| SW948_LARGE_INTESTINE | - | No mutation | No mutation |
| HH_HAEMATOPOIETIC_AND_LYMPHOID_TISSUE | - | No mutation | No mutation |
| JHOS4_OVARY | - | No mutation | No mutation |
| NCIH1915_LUNG | N/A | N/A | No mutation |
| NCIH1341_LUNG | - | No mutation | No mutation |
| JM1_HAEMATOPOIETIC_AND_LYMPHOID_TISSUE | - | No mutation | No mutation |
| DMS53_LUNG | - | No mutation | No mutation |
| BL41_HAEMATOPOIETIC_AND_LYMPHOID_TISSUE | - | No mutation | No mutation |
| GDM1_HAEMATOPOIETIC_AND_LYMPHOID_TISSUE | - | No mutation | No mutation |
| PF382_HAEMATOPOIETIC_AND_LYMPHOID_TISSUE | - | No mutation | No mutation |
| HDLM2_HAEMATOPOIETIC_AND_LYMPHOID_TISSUE | - | No mutation | No mutation |
| SUDHL5_HAEMATOPOIETIC_AND_LYMPHOID_TISSUE | - | No mutation | No mutation |
| MHHCALL2_HAEMATOPOIETIC_AND_LYMPHOID_TISSUE | - | No mutation | No mutation |
| SUPB15_HAEMATOPOIETIC_AND_LYMPHOID_TISSUE | - | No mutation | No mutation |
| GA10_HAEMATOPOIETIC_AND_LYMPHOID_TISSUE | - | No mutation | No mutation |
| DMS153_LUNG | N/A | N/A | No mutation |
| MOLT13_HAEMATOPOIETIC_AND_LYMPHOID_TISSUE | - | No mutation | No mutation |
| PL21_HAEMATOPOIETIC_AND_LYMPHOID_TISSUE | - | No mutation | No mutation |
| P12ICHIKAWA_HAEMATOPOIETIC_AND_LYMPHOID_TISSUE | - | No mutation | No mutation |
| KE37_HAEMATOPOIETIC_AND_LYMPHOID_TISSUE | - | No mutation | No mutation |
| TOLEDO_HAEMATOPOIETIC_AND_LYMPHOID_TISSUE | N/A | N/A | No mutation |
| ST486_HAEMATOPOIETIC_AND_LYMPHOID_TISSUE | - | No mutation | No mutation |
| P31FUJ_HAEMATOPOIETIC_AND_LYMPHOID_TISSUE | - | No mutation | No mutation |
| SUPT1_HAEMATOPOIETIC_AND_LYMPHOID_TISSUE | - | No mutation | No mutation |
| JURKAT_HAEMATOPOIETIC_AND_LYMPHOID_TISSUE | - | No mutation | No mutation |
| C2BBE1_LARGE_INTESTINE | - | No mutation | No mutation |
| HUCCT1_BILIARY_TRACT | BRCA2: G2044V | Missense | VUS |
| JHH2_LIVER | BRCA2: K2674N | Missense | VUS |
| COLO792_SKIN | N/A | N/A | VUS |
| IALM_LUNG | BRCA2: I1903T | Missense | VUS |
| MCC13_SKIN | BRCA2: P2532S | Missense | VUS |
| OVCAR4_OVARY | BRCA2: P2505L | Missense | VUS |
| NCIH1869_LUNG | BRCA2: P375S | Missense | VUS |
| NCIH1355_LUNG | N/A | N/A | VUS |
| MFE319_ENDOMETRIUM | N/A | N/A | VUS |
| RCC10RGB_KIDNEY | BRCA2: D1618E | Missense | VUS |
| CADOES1_BONE | BRCA2: S2616F | Missense | VUS |
| MELJUSO_SKIN | BRCA2: S755F | Missense | VUS |
| SIMA_AUTONOMIC_GANGLIA | BRCA2: V1306G | Missense | VUS |
| TE4_OESOPHAGUS | BRCA2: L3055F | Missense | VUS |
| ZR7530_BREAST | BRCA2: M2322I | Missense | VUS |
| RERFGC1B_STOMACH | N/A | N/A | VUS |
| FLO1_OESOPHAGUS | BRCA2: Y1220C | Missense | VUS |
| MDAMB361_BREAST | BRCA2: N1657S | Missense | VUS |
| CALU6_LUNG | BRCA2: E1876K | Missense | VUS |
| NCIH1648_LUNG | BRCA2: A938E | Missense | VUS |
| COLO741_SKIN | BRCA2: K382Q | Missense | VUS |
| NCIH929_HAEMATOPOIETIC_AND_LYMPHOID_TISSUE | BRCA2: R2668G | Missense | VUS |
| SISO_CERVIX | BRCA2: I411T | Missense | VUS |
| KYSE510_OESOPHAGUS | BRCA2: E1518Q, G1376E | Missense | VUS |
| COLO800_SKIN | BRCA2: H415R | Missense | VUS |
| DND41_HAEMATOPOIETIC_AND_LYMPHOID_TISSUE | BRCA2: G602E | Missense | VUS |
| IGROV1_OVARY | BRCA2: P3150T | Missense | VUS |
| NCIH28_PLEURA | BRCA2: S2590C | Missense | VUS |
| MFE296_ENDOMETRIUM | BRCA2: I332N | Missense | VUS |
| LN18_CENTRAL_NERVOUS_SYSTEM | BRCA2: N856Y | Missense | VUS |
| HUPT3_PANCREAS | BRCA2: G2044V | Missense | VUS |
| JAR_PLACENTA | N/A | N/A | VUS |
| HGC27_STOMACH | BRCA2: F2058L | Missense | VUS |
| SNU5_STOMACH | BRCA2: E2292A | Missense | VUS |
| HT_HAEMATOPOIETIC_AND_LYMPHOID_TISSUE | BRCA2: S617P | Missense | VUS |
| 22RV1_PROSTATE | BRCA2: V1810I | Missense | VUS |
| DOHH2_HAEMATOPOIETIC_AND_LYMPHOID_TISSUE | BRCA2: V3079L | Missense | VUS |
| MDAMB468_BREAST | BRCA2: M965I | Missense | VUS |
| CL34_LARGE_INTESTINE | BRCA2: V1810I | Missense | VUS |
| UACC257_SKIN | BRCA2: P2735S | Missense | VUS |
| MOLM13_HAEMATOPOIETIC_AND_LYMPHOID_TISSUE | BRCA2: Q819R | Missense | VUS |
| C33A_CERVIX | BRCA2: A3029T | Missense | VUS |
| DU145_PROSTATE | BRCA2: S2284L | Missense | VUS |
| HCC38_BREAST | BRCA2: K644R | Missense | VUS |
| SW403_LARGE_INTESTINE | BRCA2: I1851S | Missense | VUS |
| SNUC5_LARGE_INTESTINE | BRCA2: E826K | Missense | VUS |
| KATOIII_STOMACH | BRCA2: G2044V | Missense | VUS |
| DAUDI_HAEMATOPOIETIC_AND_LYMPHOID_TISSUE | BRCA2: R448C | Missense | VUS |
| NALM6_HAEMATOPOIETIC_AND_LYMPHOID_TISSUE | BRCA2: P2329L | Missense | VUS |
| NCIH661_LUNG | BRCA2: G2584V | Missense | VUS |
| LS411N_LARGE_INTESTINE | BRCA2: V831I, E3111K | Missense | VUS |
| TALL1_HAEMATOPOIETIC_AND_LYMPHOID_TISSUE | N/A | N/A | VUS |
| SNU398_LIVER | N/A | N/A | VUS |
| CCK81_LARGE_INTESTINE | BRCA2: R2973H, T1505I | Missense | VUS |
